# The NLRP3 Inflammasome Is a Pathogen Sensor for Invasive *Entamoeba histolytica* via Activation of α5β1 Integrin at the Macrophage-Amebae Intercellular Junction

**DOI:** 10.1371/journal.ppat.1004887

**Published:** 2015-05-08

**Authors:** Leanne Mortimer, France Moreau, Steve Cornick, Kris Chadee

**Affiliations:** Department of Microbiology, Immunology and Infectious Diseases, Snyder Institute for Chronic Diseases, University of Calgary, Calgary, Alberta, Canada; University of Virginia Health System, UNITED STATES

## Abstract

*Entamoeba histolytica* (*Eh*) is an extracellular protozoan parasite of humans that invades the colon to cause life-threatening intestinal and extra-intestinal amebiasis. Colonized *Eh* is asymptomatic, however, when trophozoites adhere to host cells there is a considerable inflammatory response that is critical in the pathogenesis of amebiasis. The host and/or parasite factors that trigger the inflammatory response to invading *Eh* are not well understood. We recently identified that *Eh* adherence to macrophages induces inflammasome activation and in the present study we sought to determine the molecular events upon contact that coordinates this response. Here we report that *Eh* contact-dependent activation of α_5_β_1_ integrin is critical for activation of the NLRP3 inflammasome. *Eh*-macrophage contact triggered recruitment of α_5_β_1_ integrin and NLRP3 into the intercellular junction, where α_5_β_1_ integrin underwent activation by an integrin-binding cysteine protease on the parasite surface, termed *Eh*CP5. As a result of its activation, α_5_β_1_ integrin induced ATP release into the extracellular space through opening of pannexin-1 channels that signalled through P2X_7_ receptors to deliver a critical co-stimulatory signal that activated the NLRP3 inflammasome. Both the cysteine protease activity and integrin-binding domain of *Eh*CP5 were required to trigger α_5_β_1_ integrin that led to ATP release and NLRP3 inflammasome activation. These findings reveal engagement of α_5_β_1_ integrin across the parasite-host junction is a key regulatory step that initiates robust inflammatory responses to *Eh*. We propose that α_5_β_1_ integrin distinguishes *Eh* direct contact and functions with NLRP3 as pathogenicity sensor for invasive *Eh* infection.

## Author Summary

Amebiasis caused by the enteric protozoan parasite *Entamoeba histolytica* is among the three top causes of death from parasitic infections worldwide, as a result of amebic colitis (dysentery) and liver or brain abscess. When *Eh* invades the intestinal barrier and contacts host tissue there is a profound inflammatory response, which is thought to drive the disease. One of the central outstanding questions has been how the immune response is escalated at sites of invasion. Adherence of the parasite to host cells has long been appreciated in the pathogenesis of amebiasis, but was never considered as a “cue” that host cells use to detect *Eh* and initiate host defense. Here we introduce the idea, and demonstrate, that an intercellular junction forms between *Eh* and host cells upon contact that engages the NLRP3 inflammasome. The NLRP3 inflammasome belongs to a group of “danger” sensors that are uniquely designed to rapidly activate highly inflammatory host defenses. In this work, we identified a surface receptor on macrophages that normally functions in adhesion and polarization recognizes a protein on the outer surface of *Eh*. Intriguingly, *Eh* also secretes this protein. However, the full activation of the surface receptor leading to inflammasome activation only occurs when the *Eh* protein is immobilized on the parasite surface. Thus, we uncovered a molecular mechanism though which host cells distinguish direct contact, and therefore recognize parasites that are immediately present in the tissue, to mobilize a highly inflammatory response. We believe this concept is central to understanding the biology of amebiasis.

## Introduction

*Entamoeba histolytica*, the etiologic agent of amebiasis, is a protozoan parasite of the human colon that colonizes about 10% of the world’s population resulting in 10^6^ deaths/year and is endemic in areas that lack adequate water sanitation [1]. Infection is acquired by ingesting cysts that release trophozoites that feed and replicate in the colon [2]. The majority of infections remain in the lumen and are tolerated without disease. For unknown reasons *Eh* occasionally breaches innate mucosal barriers and invades the lamina propria and submucosa where the parasite can further disseminate through the portal circulation and infect the liver. When *Eh* invades, there is a florid inflammatory response, components of which are thought to exacerbate the disease [2]. Currently, we lack an understanding of normal immune mechanisms that trigger this inflammatory response.

One of the central outstanding questions has been how the immune response is escalated at sites of invasion. In this regard, adherence of the parasite to host cells has long been appreciated in the pathogenesis of amebiasis, but has been overlooked as an event that itself initiates host defense and inflammation [3, 4]. During a microbial encounter the innate immune system uses a variety of cues to distinguish both the organism and the level of danger that that organism presents in order to respond appropriately so that robust host defenses that cause significant bystander damage are only triggered when pathogenic threats are severe. In this manner, a direct interaction between host cells and *Eh* should signify the presence of an immediate infection. In turn, the immune response should be rapidly scaled-up precisely at locations where active infections are detected to eliminate and prevent further spread of the parasite. Therefore, how the innate immune system directly recognizes *Eh* and how this scenario initiates and shapes host defense is critical to understand the basis of the host response and the pathogenesis of amebiasis. To address this issue, it needs to be appreciated that *Eh* are large, between 20–60 μM in diameter and are too big to be phagocytosed by innate immune cells. As *Eh* remain extracellular throughout infection, host cells acquire information about the immediate presence of *Eh* at points of membrane contact with trophozoites. We think this interaction is critical in understanding the pathogenesis of amebiasis.

Macrophages are thought to be crucial in the innate immune response to invasive *Eh* by killing the parasite directly and by driving an inflammatory response that recruits additional immune cell help to combat the infection [5, 6]. High mobility and the ability to form dynamic intercellular contacts are central to the macrophage immune-surveillance system enabling them to survey their environment for microorganisms [7]. From the onset of contact macrophages gather information about the nature of a target by exploring it’s surface by engagement of surface receptors and interactions with the plasma membrane. This leads to the recruitment and clustering of receptors at points of contact to specific molecules on the target surface, and selective activation of signaling pathways. We recently identified that direct *Eh* contact with macrophages induces inflammasome activation, though we did not identify the type of inflammasome involved [8]. Inflammasomes are a group of intracellular multi-protein complexes that link specific pro-inflammatory stimuli to the activation of caspase-1 [9]. Active caspase-1 in turn initiates highly potent inflammatory responses by cleaving the intracellular pro-forms of interleukin (IL)-1β and IL-18 into their active forms and mediates their release, along with a number of other pro-inflammatory mediators, by a secretion event that remains to be defined. We determined that the major *Eh* surface adhesin, the Gal-lectin, provides an “adhesive” signal that enables intercellular contacts with macrophages to form, which was critical for activating the inflammasome [8]. Thus, if we blocked *Eh*-Gal-lectin with monoclonal antibodies that specifically target regions involved in adhesion, or if we added D-galactose to disrupt binding to galactose moieties on unknown macrophage receptors, or if we added soluble *Eh*-Gal-lectin to competitively inhibit *Eh* adherence, inflammasome activation as well as intercellular contacts were abolished [8]. The surface receptors on macrophages that ligate *Eh*-Gal-lectin are currently unknown and remain an area of interest. However, in the present study we explored the possibility that contact provides a platform through which other *Eh* surface molecules interact to coordinate inflammasome activation. Here we show that an *Eh* cysteine protease, termed *Eh*CP5 that like *Eh*-Gal-lectin is expressed on the trophozoite surface and is secreted, is necessary for contact-dependent inflammasome activation.

We found that α_5_β_1_ integrin, a surface receptor critically involved in cell adhesion, polarization and migration is recruited into sites of contact and is highly activated by surface-bound *Eh*CP5 through its integrin-binding RGD sequence and proteolytic activity. When soluble *Eh*CP5 was presented to macrophages it ligated α_5_β_1_ integrin but did not enhance activation. However, when an intercellular junction was formed, *Eh*CP5 on the trophozoite surface stimulated rapid and robust activation of α_5_β_1_ integrin. This event triggered macrophages to produce an extracellular burst of ATP through opening surface pannexin-1 (Panx1) channels that were activated by α_5_β_1_ integrin signaling. Subsequently, ATP delivered a critical stimulus to activate the NLRP3 inflammasome. Thus, direct sensing of *Eh* occurs by engagement of α_5_β_1_ integrin at the intercellular junction where NLRP3 functions as a pathogenicity sensor for invasive *Eh*.

## Results

### NLRP3 recruitment into contact sites with *E*. *histolytica* is required to activate the NLRP3 inflammasome

**Fig 1:**
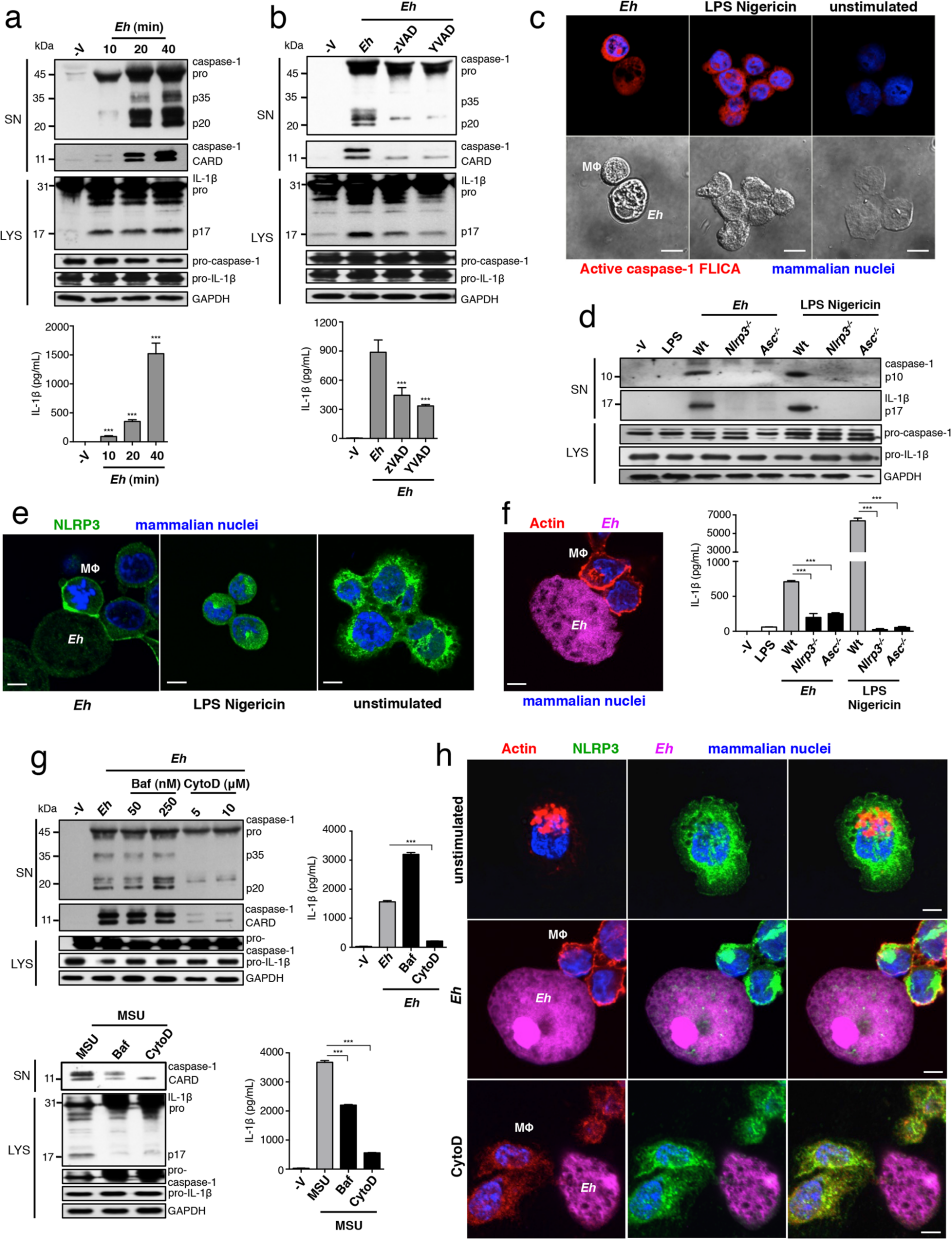
*E*. *histolytica* activates the NLRP3 inflammasome upon contact. PMA-differentiated THP-1 macrophages (**a-c, e-h**) and BMDM (**d**) treated for 30 min or the indicated time with live *Eh* at a 1:30 ratio, without inhibitors (**a, c, d**), or with the addition of 100 μM zVAD-fmk (**b**), 100 μM YVAD-fmk (**b**), bafiolmycin A (**g**) or cytochalasin D (**g**) to the cultures before stimulation. Secretion and processing of IL-1β was determined by immunoblot and enzyme-linked immunosorbent assay (**a, b, d, g**). Secretion of active caspase-1 cleavage products (p20 and CARD, human; p10, mouse) was determined by immunoblot (**a, b, d, g**). (**c**) Caspase-1 activation after 30 min with LPS Nigericin or upon contact with *Eh* in which active caspase-1 was stained with YVAD-FLICA, red; mammalian nuclei, blue (*Eh* nuclei are not stained). (**e, h**) Localization of endogenous NLRP3 after stimulation with *Eh* or LPS Nigericin. (**f, h**) Localization of F-actin after *Eh* stimulation; CFSE-labeled *Eh*, purple. *Eh*, *E*. *histolytica*; Mϕ, macrophages; Baf, bafiolmycin A; CytoD, cytochalasin D; MSU, monosodium urate; SN, supernatants; LYS, cell lysates. Scale bars, 10 μM. Data are representative of three separate experiments (error bars SEM). ***P < 0.005.

In unstimulated macrophages, caspase-1 exists as a partially active pro-enzyme of 45–55 kDa, and after recruitment to an active inflammasome platform undergoes sequential autoproteolysis to become fully active, and is secreted from the cell. *Eh* placed in direct contact with macrophages rapidly induced caspase-1 and IL-1β activation, as caspase-1 and IL-1β began to appear in the culture media within 10 min (**Fig 1A–1C**). Note that the cleavage patterns of caspase-1 result from different steps of caspase-1 processing and differences in where pro-caspase-1 and fully active caspase-1 cleave caspase-1 molecules [10]. The pro-enzyme is first cleaved into the C-terminal p10 subunit and a more active p35 fragment composed of the p20 subunit and the N-terminal CARD [10]. Cleavage of the p35 fragment separates the CARD and p20 subunit, and the p10 and p20 subunits form a fully active heterodimer [10]. We used two different human caspase-1 antibodies to assess caspase-1 activation in response to *Eh*: one that detects the p20, p35 and pro-caspase-1 (top immunoblot) and another that detects the cleaved CARD domain (bottom immunoblot).

We have previously shown that intact ameba directly in contact with macrophages triggers inflammasome activation, while soluble components of whole *Eh*, even after extended culture, do not [8]. We observed that intercellular contacts formed between *Eh* and macrophages were dependent on the major *Eh* surface adhesin, the Gal-lectin, and that Gal-lectin-mediated contact was necessary for inflammasome activation. In contrast, soluble *Eh*-Gal-lectin did not stimulate the inflammasome and competitively inhibited *Eh*-contact-from triggering inflammasome activation [8]. These data suggested the inflammasome involved is a selective sensor for *Eh* contact and furthermore, that signaling at the point of contact critically regulates inflammasome activation.

To determine which type of inflammasome was involved we used pharmacological inhibitors of the NLRP3 inflammasome and found these abrogated *Eh*-induced caspase-1 and IL-1β cleavage and secretion in human THP-1 macrophages (**S1A–S1C Fig**). To verify whether the NLRP3 inflammasome was required, we performed siRNA knockdown of NLRP3 and the adaptor ASC in human macrophages, which inhibited the cleavage and secretion of caspase-1 and IL-1β (**S2A and S2B Fig**). Similarly, caspase-1 and IL-1β cleavage and secretion did not occur in *nlrp3* ^*-/-*^ and *asc* ^*-/-*^ BMDMs stimulated with *Eh* (**Fig 1D**). These data established that NLRP3 is the inflammasome activated by *Eh* contact. Interestingly, we observed that NLRP3 redistributed to the cell periphery and intensely localized at *Eh*-macrophage junctions (**Fig 1E**). This localization pattern was dramatically different from unstimulated macrophages, where NLRP3 was dispersed throughout the cytoplasm, and from cells stimulated with soluble NLRP3 inflammasome activators LPS and nigericin, where NLRP3 organized in cytoplasmic puncta (**Fig 1E**). These data indicate that attachment of *Eh* to macrophages triggers recruitment of NLRP3 to the microenvironment near the point of contact and that this is a critical step in the activation of the NLRP3 inflammasome.

Macrophages undergo dramatic cytoskeletal reorganization upon contact with *Eh* where F-actin accumulates at the intercellular junction similar to NLRP3 (**Fig 1F**). This led us to ask whether actin remodeling was required for *Eh* to recruit and induce NLRP3 activation. Addition of cytochalasin D, an inhibitor of F-actin inhibited caspase-1 and IL-1β cleavage and secretion in response to *Eh* (**Fig 1G**). Several NLRP3 stimuli require actin-dependent phagocytosis to initiate phagolysosomal damage [11–13], though we suspected this pathway was not involved because *Eh* induced caspase-1 and IL-1β release within 10 min (**Fig 1A**), and phagolysosomal-induced activation of NLRP3 takes several hours [11–13]. To confirm this we cultured macrophages in bafilomycin to block endosome acidification, which is essential for phagolysosomal damage to activate NLRP3. While bafilomycin inhibited inflammasome activation in response to monosodium urate, which is phagocytosis-dependent, it had no effect on *Eh*-induced activation (**Fig 1G**). Microtubules have been reported to universally regulate the activation of NLRP3 by relocating mitochondria into the proximity of NLRP3 on the endoplasmic reticulum [14]. However, inhibition of tubulin polymerization with colchicine did not suppress NLRP3 inflammasome activation by *Eh*. Instead, it significantly enhanced activation, indicating that the microtubule system is not required for *Eh*-induced NLRP3 activation (**S3 Fig**). Furthermore, cytochalasin D abrogated NLRP3 recruitment into contact sites (**Fig 1H**), indicating these are signaling-rich locations that regulate NLRP3 recruitment through an actin-dependent pathway. Based on this, we hypothesized that NLRP3 activation was regulated by a surface receptor in the intercellular junction that is connected to the actin cytoskeleton.

### α_5_β_1_ integrin is activated by *Eh* contact via a surface-bound-integrin-binding *E*. *histolytica* cysteine protease

We were interested in host receptors that could function as sensors for distinguishing contact with the *Eh* surface. To this end, we recently identified that *Eh* cysteine protease 5 (*Eh*CP5), a secreted cysteine protease that associates on the membrane of *Eh*, activates epithelial α_v_β_3_ integrin via an RGD sequence [15]. Integrins are transmembrane receptors that connect with the cytoskeleton to control cell adhesion and processes dependent on cytoskeletal remodelling [16]. In addition to being regulated by ligand binding, their level of activation depends on receptor density and the size of the contact surface with which they interact [16]. These features could allow integrins to distinguish contact with a microbial surface if a ligand was present at the cell-cell interface.

**Fig 2:**
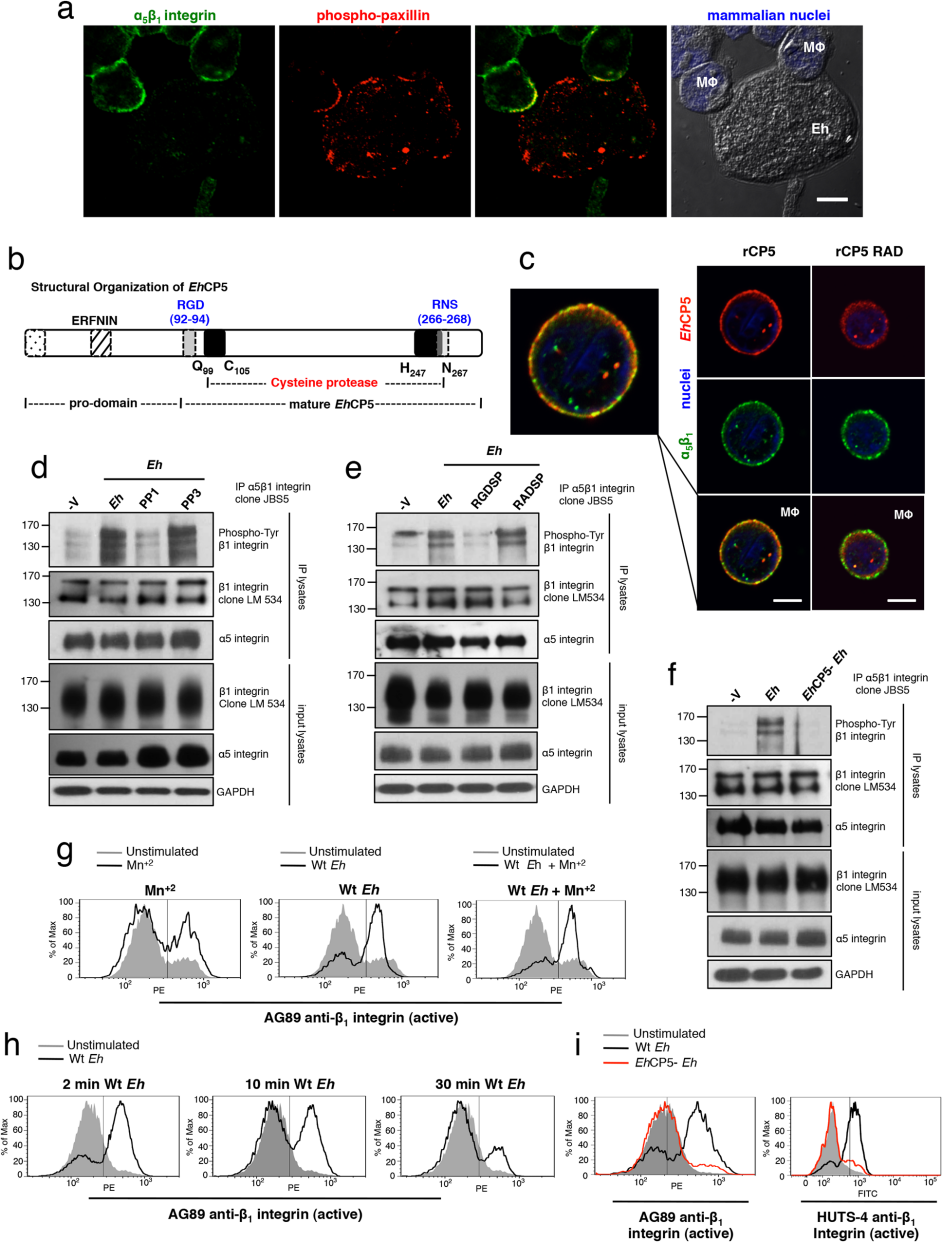
α_5_β_1_ integrin is activated by *Eh* contact via a surface-bound-integrin-binding *Eh* cysteine protease. (**a**) Localization of α_**5**_β_**1**_ integrin (green) and phosphorylated (active)-paxillin (red) in PMA-differentiated macrophages at sites of *Eh* contact; mammalian nuclei, blue (*Eh* nuclei are not labeled). (**b**) Diagram showing structural organization of *Eh*CP5. (**c**) Subcellular distribution of *Eh*CP5 (red) and α_**5**_β_**1**_ integrin (green) after THP-1 macrophages were cultured with rCP5 (left) or rCP5 in which the RGD site was mutated to RAD (right); nuclei, blue. (**d-f**) Immunoblot analysis of β_**1**_ integrin tyrosine phosphorylation (clone PY20) of anti-α_**5**_β_**1**_ integrin immunoprecipitates from PMA-differentiated THP-1 macrophages stimulated with Wt *Eh* (**d-f**), with PP1 or PP3 control (**d**), or blocking peptide RGDSP or RADSP control (**e**) added to cultures 10 min before stimulation, or *Eh*CP5- *Eh* (**f**). (**g-i**) Activation status of β_**1**_ integrin in PMA-differentiated THP-1 macrophages stimulated with Wt (**g-i**), or *Eh*CP5- *Eh* (**i**) evaluated with anti-β_**1**_ integrin mAbs, AG89 (**g-i**) and HUTS-4 (**i**) that recognize the active conformation specific epitope of β_**1**_ integrin. Scale bars, 10 μM. Data are representative of two (**a, c, i**) or three (**d-h**) separate experiments.

Confocal immunofluorescence revealed that α_5_β_1_ integrin and phosphorylated-paxillin, a scaffold protein that is recruited to and phosphorylated at sites where integrins are active, were highly concentrated at *Eh*-macrophage contacts (**Figs 2A and S4**). Further analysis revealed that *Eh*CP5 contains an RNS accessory sequence, which in human fibronectin synergizes with the RGD to bind and activate α_5_β_1_ integrin [17] (**Fig 2B**). Indeed, we found that recombinant *Eh*CP5 (r*Eh*CP5) immunoprecipitated with α_5_β_1_ integrin (**S5 Fig)** and co-localized with α_5_β_1_ integrin on the surface of macrophages (**Fig 2C**). In contrast, r*Eh*CP5 in which the RGD was mutated to RAD did not immunoprecipitate or co-localize with α_5_β_1_ integrin on the surface of macrophages (**Figs 2C and S5**), showing that *Eh*CP5 interacted with α_5_β_1_ integrin in an RGD-dependent manner. Note that r*Eh*CP5 with the mutated RGD motif was still taken up by macrophages, likely by pinocytosis. However, it did not label nor co-localize with α_5_β_1_ integrin on macrophage surfaces (**Fig 2C**).

We then sought to determine the activation status of α_5_β_1_ integrin upon *Eh* contact. The cytoplasmic domain of β_1_ integrin contains two tyrosine residues in conserved NPXY and NXXY motifs that are phosphorylated by Src family kinases (SFK) upon α_5_β_1_ integrin activation and are essential for recruiting adaptors that facilitate coupling of the integrin to the actin cytokskeleton and for maintaining it in an active state [18]. Immunoprecipitation of α_5_β_1_ integrin followed by anti-phospho-tyrosine immunoblot of THP-1 macrophages stimulated with *Eh* detected 160 kDa, 140 kDa and 120 kDa phospho-bands reported for β_1_ integrin [19] (**Fig 2D**). *Eh*-induced phosphorylation of the β_1_ subunit occurred in an SFK-dependent manner, as phosphorylation was inhibited by PP1 but not the inactive analog PP3 (**Fig 2D**). To test whether phosphorylation was dependent on an RGD-containing ligand, we used a soluble RGDSP peptide, which competes out binding and activation of RGD-binding integrins. The RGDSP peptide, but not the RADSP control inhibited *Eh*-induced phosphorylation of the β_1_ subunit in α_5_β_1_ integrin immunoprecipitates (**Fig 2E**).

To determine whether *Eh*CP5 induced α_5_β_1_ integrin phosphorylation, we stimulated macrophages with *Eh* that were stably silenced for *Eh*CP5 (hereafter designated *Eh*CP5^-^ *Eh*) and found that, in contrast to wildtype (Wt) *Eh*, *Eh*CP5^-^ *Eh* did not induce phosphorylation of the β_1_ subunit (**Fig 2F**). We further evaluated the activation status of the β_1_ subunit using two monoclonal antibodies AG89 and HUTS-4, which recognize the active conformation specific epitope of β_1_ integrin and performed flow cytometry to evaluate whether *Eh* enhanced its expression. The active epitope, which was basally expressed on unstimulated THP-1 macrophages, was rapidly and transiently increased upon addition of Wt *Eh* and was not further enhanced by the addition of Mn^+2^ which stabilizes the β_1_ subunit active conformation (**Fig 2G–2I**), whereas *Eh*CP5^-^ *Eh* did not induce the active β_1_ epitope (**Fig 2I**). Interestingly, though soluble *Eh*CP5 interacted with α_5_β_1_ integrin, it neither induced phosphorylation of α_5_β_1_ integrin nor expression of the active β_1_ epitope. Together, these data showed that α_5_β_1_ integrin was specifically activated by *Eh*CP5 upon contact and suggested that α_5_β_1_ integrin may sense *Eh* adhesion that activates the NLRP3 inflammasome.

### α_5_β_1_ integrin activation and *Eh*CP5 are required for *E*. *histolytica* to activate the NLRP3 inflammasome

**Fig 3:**
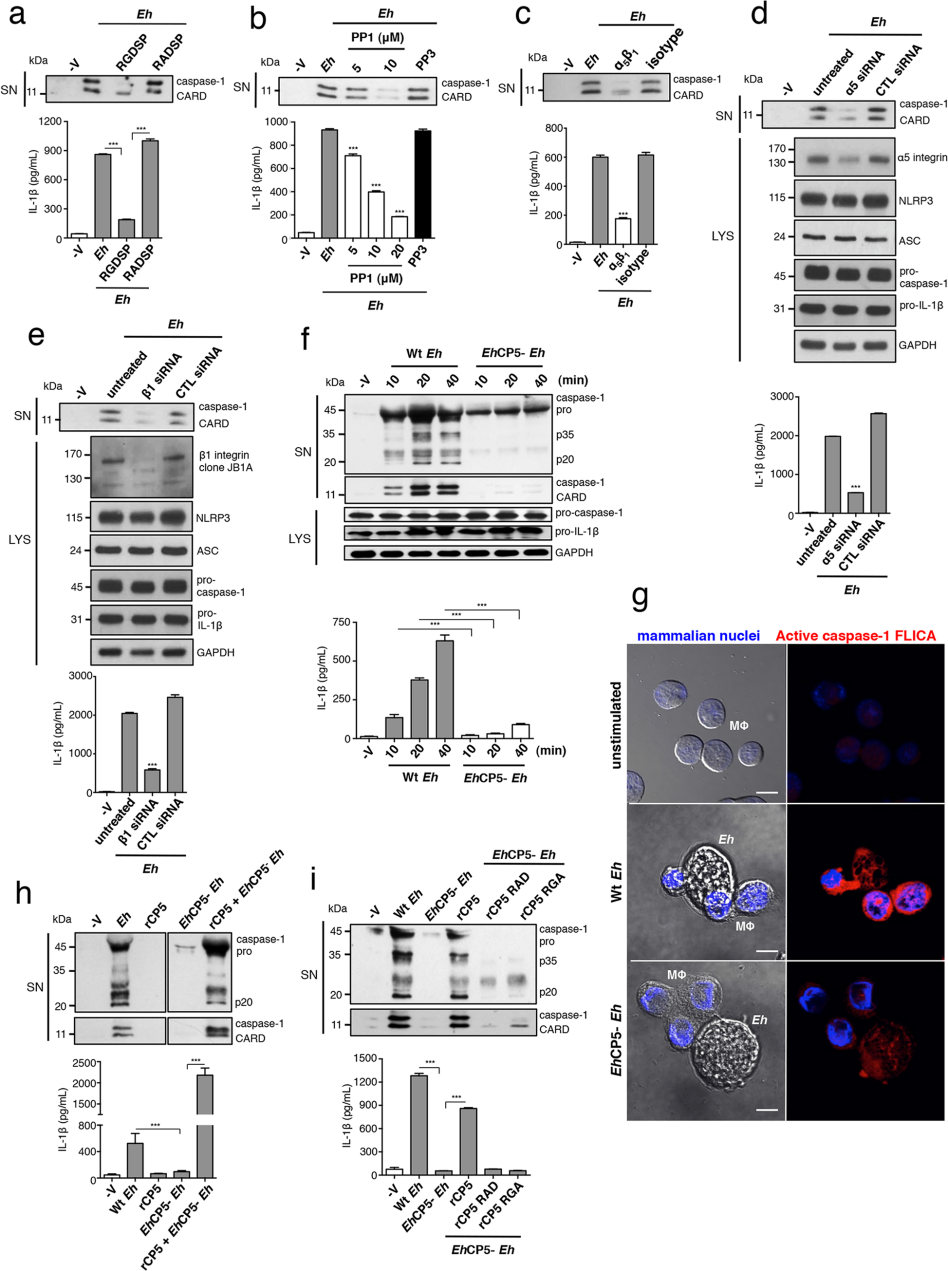
α_5_β_1_ integrin activation and *Eh*CP5 are required for *E*. *histolytica* (*Eh*) to activate the NLRP3 inflammasome. (**a-f, h, i**) Immunoblot analysis of secreted active caspase-1 cleavage products and IL-1β enzyme-linked immunosorbent assay of PMA-differentiated THP-1 macrophages stimulated for 30 min or the indicated time with live *Eh* at a 1:30 ratio, without inhibitors (**f, h, i**), or with the addition of integrin RGD blocking peptide RGDSP or RADSP control (50 μM) (**a**), or SFK inhibitor PP1 or PP3 control (**b**), or a function blocking antibody to α_**5**_β_**1**_ integrin (10 μg mL^-1^) (**c**) added to cultures 10 min before stimulation, or following siRNA knockdown of α_**5**_ integrin (**d**) and β_**1**_ integrin (**e**). (**d, e**) Immunoblot analysis of α_**5**_ integrin (**d**) and β_**1**_ integrin (**e**) and inflammasome molecules following siRNA knockdown. (**f, h, i**) Macrophages stimulated with Wt or *Eh*CP5- *Eh* with the addition of rCP5 (1 μg mL^-1^) (**h, i**), or rCP5 in which the RGD site was mutated to RAD or RGA (rCP5 RAD, rCP5 RGA respectively; 1 μg mL^-1^) (**i**) to cultures. (**g**) Caspase-1 activation upon contact with Wt or *Eh*CP5- *Eh* in which active caspase-1 was stained with YVAD-FLICA, red; mammalian nuclei, blue (*Eh* nuclei are not stained). Scale bars, 10 μM. Data are representative of two (**g, h**) or three (**a-f, i**) separate experiments (error bars SEM). ***P < 0.005.

We next addressed whether α_5_β_1_ integrin is required for *Eh* to trigger the NLRP3 inflammasome. First we tested if activation of an RGD-binding integrin was required. Addition of soluble RGDSP, but not the RADSP control, inhibited caspase-1 and IL-1β activation by *Eh* (**Fig 3A**). SFKs are activated upon integrin ligation during the proximal signaling cascade [18]. Addition of the SFK inhibitor PP1, but not the inactive analog PP3, dose-dependently blocked caspase-1 and IL-1β activation by *Eh* (**Fig 3B**). These data showed that signaling of an RGD-binding integrin is required for *Eh* to activate the NLRP3 inflammasome. We therefore investigated whether α_5_β_1_ integrin was involved. THP-1 macrophages express three RGD-binding integrins: α_5_β_1_, α_4_β_1_ and α_v_β_3_ [20–22]. Function blocking antibodies to the β_1_ subunit, α_5_ subunit and to α_5_β_1_ integrin, but not to the α_4_ subunit or to α_v_β_3_ integrin, abrogated caspase-1 and IL-1β secretion (**Figs 3C, S6A and S6C**). We further confirmed these results with siRNA knockdown of either β_1_ or α_5_ integrin in THP-1 macrophages, which did not alter expression of the NLRP3 inflammasome components (**Fig 3D and 3E**). Knockdown of either subunit prevented caspase-1 and IL-1β activation (**Fig 3D and 3E**). Therefore, α_5_β_1_ integrin is required for *Eh* to trigger the NLRP3 inflammasome.

As we identified *Eh*CP5 as a ligand for activating α_5_β_1_ integrin, these results predicted that *Eh*CP5 would be necessary for *Eh* to activate the inflammasome. We measured caspase-1 and IL-1β activation in macrophages stimulated with *Eh*CP5^-^ *Eh* and found that in contrast to Wt *Eh*, *Eh*CP5^-^ *Eh* did not activate the inflammasome (**Figs 3F, 3G and S7A**). There was no difference in caspase-1 and IL-1β activation when macrophages were stimulated with the vector control strain (**S7B Fig**; see Material and Methods for strain designation). Though soluble r*Eh*CP5 alone did not trigger inflammasome activation, which is consistent with the fact that soluble r*Eh*CP5 did not activate α_5_β_1_ integrin, when it was simultaneously applied with *Eh*CP5^-^ *Eh*, r*Eh*CP5 restored caspase-1 and IL-1β activation (**Fig 3H**). This is likely due to *Eh*CP5 being able to associate on the membrane of *Eh* after it is secreted, still allowing *Eh*CP5 and α_5_β_1_ integrin to interact and reorganize into the junction and therefore to permit α_5_β_1_ integrin activation. To confirm that the RGD sequence was critical for *Eh*CP5 to activate NLRP3, r*Eh*CP5 in which the RGD sequence was mutated to RAD or RGA were tested for their ability to restore inflammasome activation to *Eh*CP5^-^ *Eh*. Unlike Wt r*Eh*CP5, neither r*Eh*CP5-RAD nor r*Eh*CP5-RGA restored caspase-1 and IL-1β activation to *Eh*CP5^-^ *Eh* (**Fig 3I**). Thus, *Eh*CP5 interacts with α_5_β_1_ integrin in an RGD-dependent manner to trigger activation of the NLRP3 inflammasome.

### ATP release via α_5_β_1_ integrin is required for NLRP3 inflammasome activation by *Eh*

**Fig 4:**
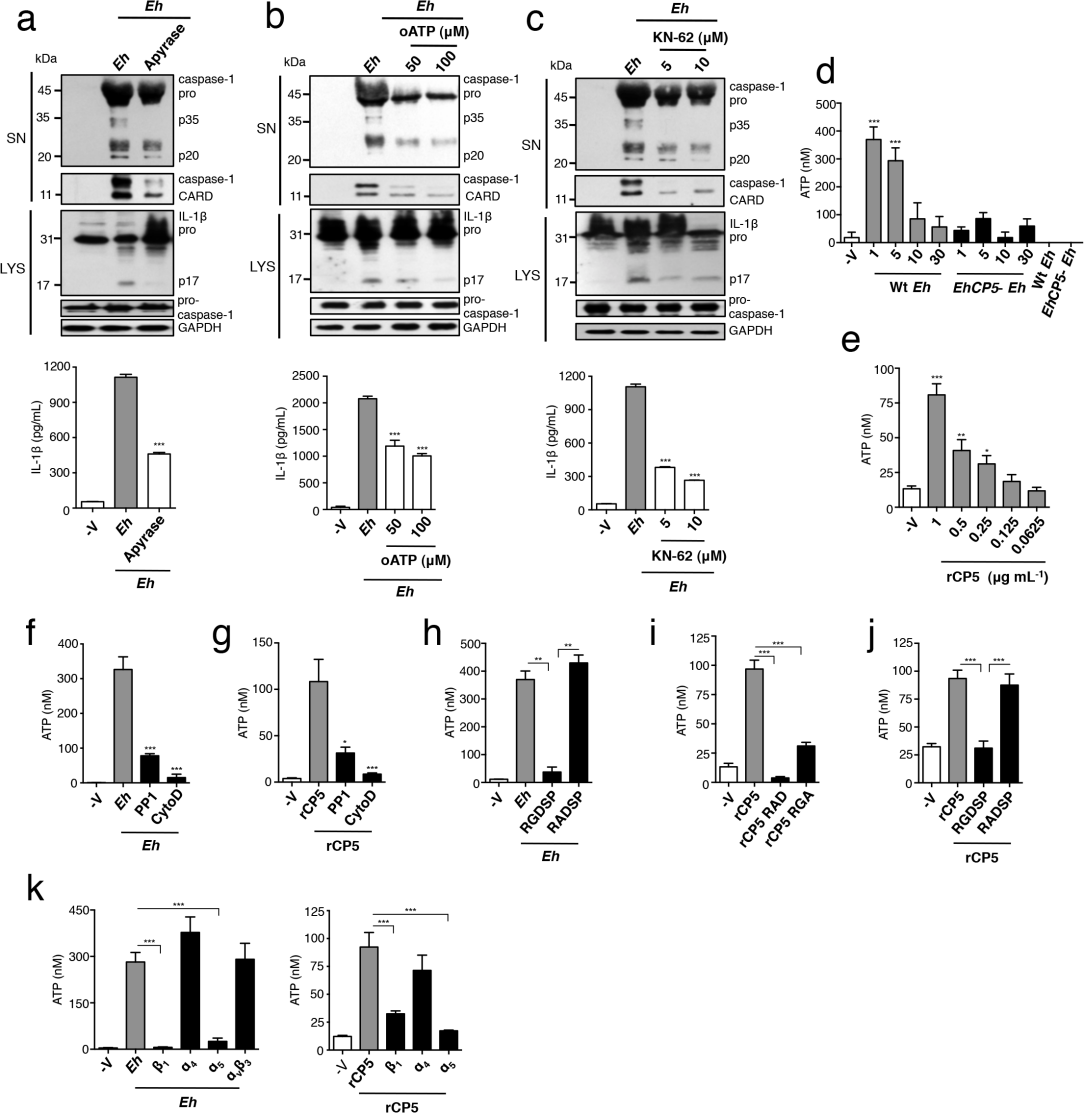
ATP release via α_5_β_1_ integrin is required for *E*. *histolytica* (*Eh*) to activate the NLRP3 inflammasome. (**a, b, c**) Immunoblot analysis of active caspase-1 and IL-1β cleavage products and IL-1β enzyme-linked immunosorbent assay of PMA-differentiated THP-1 macrophages stimulated for 30 min with Wt *Eh* with the addition of apyrase (30 U mL^-1^) (**a**), or P2X_**7**_R inhibitors oATP (50–100 μM) (**b**), or KN-62 (5–10 μM) (**c**). (**d-k**) ATP release from PMA-differentiated THP-1 macrophages stimulated for 1 min with or the indicated time with a 1:30 ratio of Wt *Eh* (**d**), *Eh*CP5- *Eh* (**d**), or rCP5 (**e**), rCP5 RAD (**i**), rCP5 RGA (1 μg mL^-1^ or indicated concentration) (**i**) without inhibitors or with PP1 control (10 μM) (**f, g**), or cytochalasin D (10 μM) (**f, g**), or RGDSP peptide or RADSP control (**h, j**) (25 μM), or function blocking antibodies to β_**1,**_ α_**4**_, and α_**5**_ integrin, α_**v**_β_**3**_ integrin (10 μg mL^-1^) (**k**) added to cultures 10 min before stimulation or 45 min before stimulation for cytochalasin D. Data are representative of two (**d**, **h-k**) or three (**a-c**, **e-g**) separate experiments (error bars SEM). ***P < 0.005, *P < 0.05. oATP, oxidized adenosine triphosphate.

We next sought to identify how α_5_β_1_ integrin regulates NLRP3 activation. In our hands the kinetics and strength of caspase-1 and IL-1β activation in response to *Eh* is similar to ATP stimulation of NLRP3. ATP is a well-established physiological stimulus of the NLRP3 inflammasome that signals through the ATP-gated P2X_7_ receptor (P2X_7_R), and it was of interest to us that P2X_7_R complexes with integrins, as it suggested that α_5_β_1_ integrin may regulate this pathway [23]. We therefore investigated whether ATP-P2X_7_R signaling was involved in NLRP3 activation upon *Eh* contact. We cultured macrophages with apyrase, which hydrolyses ATP to AMP and PPi, and this reduced caspase-1 and IL-1β activation in response to *Eh* (**Fig 4A**). Similarly, specific antagonists of the P2X_7_R, oATP and KN-62, inhibited caspase-1 and IL-1β activation, indicating an essential role for ATP-P2X_7_R signaling (**Fig 4B and 4C**). We then measured extracellular ATP in *Eh*-macrophage cultures. Naïve macrophages (negative control) released low levels of ATP whereas *Eh* released none (**Fig 4D**). However, when the two were placed together, we observed a rapid spike of extracellular ATP that diminished in a time-dependent fashion due to ubiquitously expressed ecto-ATPases that rapidly degrade extracellular ATP [24] (**Fig 4D**). *Eh* did not cause macrophage lysis that could account for the rapid burst of ATP, as there was no LDH release or trypan blue staining, even up to 30 minutes. We considered whether ATP release was induced by *Eh*CP5 activation of α_5_β_1_ integrin and made a series of striking observations. Our first observation was that *Eh*CP5^-^ *Eh* did not stimulate ATP release (**Fig 4D**). We found that soluble r*Eh*CP5 dose-dependently induced ATP, though the level was much lower than with intact *Eh* (**Fig 4E**). Therefore, *Eh*CP5 is essential for triggering ATP, which it can induce directly from macrophages, but its activity is enhanced by *Eh* contact. This pointed to a role for formation of actin-dependent contacts in regulating ATP release. When we inhibited actin polymerization in macrophages by adding cytochalasin D, ATP release was completely abolished in response to both *Eh* and r*Eh*CP5 (**Fig 4F and 4G**).

Next we tested if ATP release required α_5_β_1_ integrin activation. Addition of soluble RGDSP and the SFK inhibitor PP1, but not soluble RADSP or PP3 controls, abolished ATP secretion in response to *Eh*, demonstrating that activation of an RGD-binding integrin was essential (**Fig 4F and 4H**). Consistent with this, the integrin-binding site of r*Eh*CP5 was critical to stimulate ATP, as neither r*Eh*CP5-RAD nor r*Eh*CP5-RGA induced ATP release and soluble RGDSP blocked ATP by r*Eh*CP5 (**Fig 4I and 4J**). Furthermore, inhibition of SFKs by PP1 abrogated ATP release by r*Eh*CP5 (**Fig 4G**). Thus, although we did not detect α_5_β_1_ integrin phosphorylation or expression of the active β_1_ epitope in response to soluble r*Eh*CP5, a low level of integrin activation occurred that caused some ATP release. Finally, to establish that α_5_β_1_ integrin was essential for releasing ATP we cultured macrophages with function-blocking antibodies to the three RGD-binding integrins expressed on THP-1 macrophages, α_5_β_1_, α_4_β_1_ and α_v_β_3_. Similar to the results obtained for inflammasome activation, only antibodies that blocked the α_5_ and β_1_ subunits, and not α_4_ subunit and α_v_β_3_, inhibited ATP release in response to both *Eh* and r*Eh*CP5 (**Fig 4K**). Activation of α_5_β_1_ integrin by *Eh*CP5 therefore, induced the rapid release of ATP into the extracellular space that was critical to activate the NLRP3 inflammasome. Furthermore, we conclude that a high threshold of α_5_β_1_ activation is reached only by contact, guaranteeing that a high concentration of extracellular ATP only occurs when *Eh* is directly present.

### Panx-1 channels release ATP in response to *E*. *histolytica*

**Fig 5:**
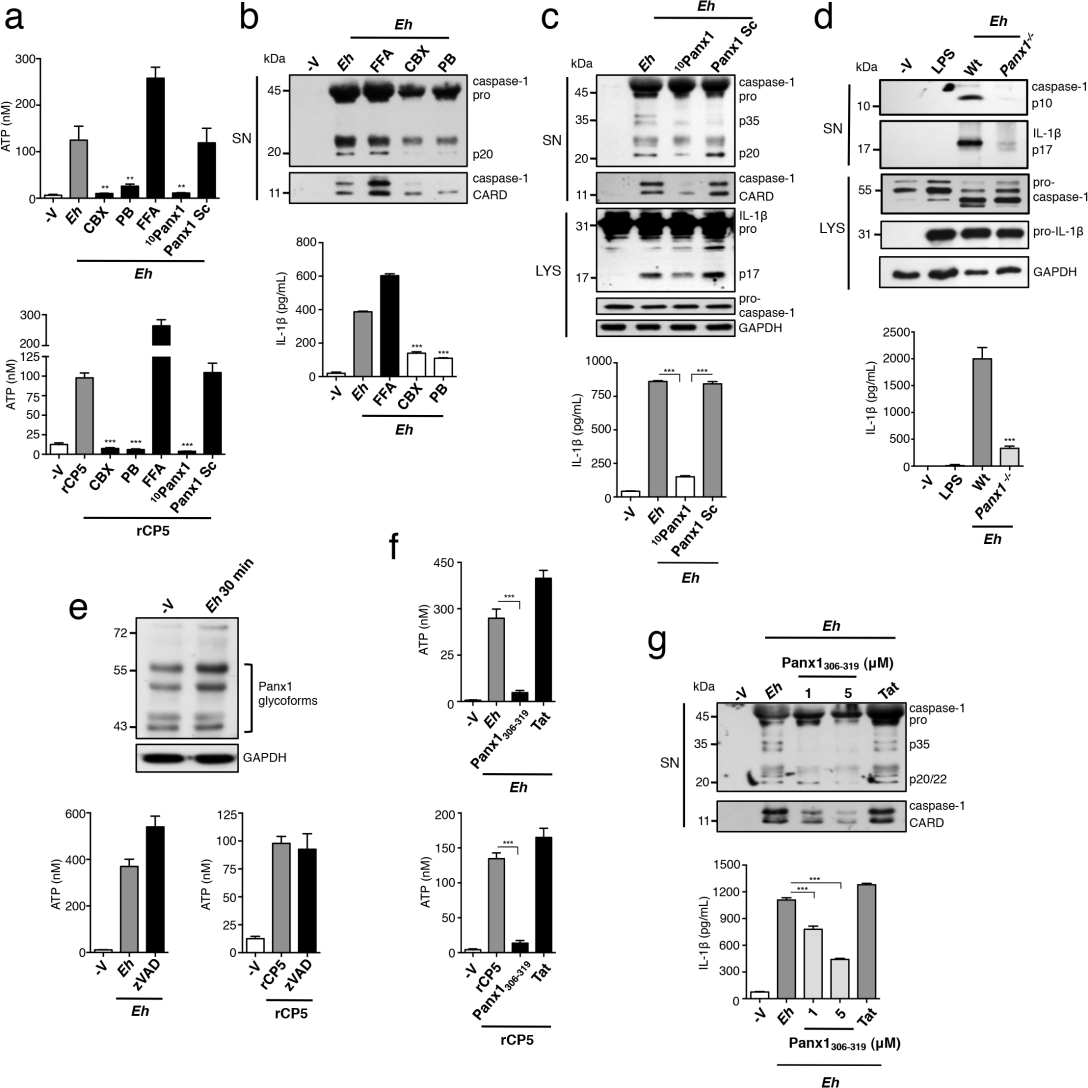
Panx-1 channels release ATP in response to *E*. *histolytica* (*Eh*). (**a, e, f**) ATP release from PMA-differentiated THP-1 macrophages stimulated for 1 min with a 1:30 ratio of Wt *Eh* or rCP5 (1 μg mL^-1^) with connexin/pannexin channel dual inhibitor carbenoxolone (CBX, 100 μM) (**a**), or pannexin channel inhibitor probenecid (PB, 250 μM) (**a**), or connexin channel inhibitor flufenamic acid (FFA, 100 μM) (**a**), or Panx1-peptide inhibitor ^10^Panx1 (500 μM) or scrambled control (500 μM) (**a**), or zVAD-fmk (100 μM) (**e**), or a mimetic peptide corresponding to an SFK-like consensus sequence of Panx1, Panx1_**306-319**_ (1 μM) or Tat control (1 μM) (**f**) added to cultures 10 min before stimulation or 45 min before stimulation for zVAD. (**c-d, g**) Immunoblot analysis of active caspase-1 and IL-1β cleavage products and IL-1β enzyme-linked immunosorbent assay of PMA-differentiated THP-1 macrophages (**c, b, g**) or BMDMs (**d**) stimulated for 30 min with a 1:30 ratio of Wt *Eh* without inhibitors (**d**) or with addition of CBX (**b**), PB (**b**), FFA (**b**), ^10^Panx1 (**c**) or Panx1_**306-319**_ (**g**) as in (**a**) and (**f**), respectively. (**e**) Analysis of Panx1 expression following stimulation of THP-1 macrophages for 30 min with *Eh*. Data are representative of two (**a, f, d**) and three separate (**b, c, g, e**) experiments (error bars SEM). ***P < 0.005.

ATP is conducted into the extracellular space by non-junctional (hemi)-channels of the pannexin and connexin families [25]. To test whether *Eh* induced ATP release through either of these channels we applied inhibitors to macrophage cultures. We found that carbenoxolone (CBX) and probenecid (PB), a dual antagonist of pannexin and connexin channels and a pannexin-specific antagonist, respectively completely abolished ATP release in response to *Eh* and r*Eh*CP5 (**Fig 5A**). In contrast, the connexin-specific inhibitor flufenamic acid (FFA) did not block ATP release; in fact FFA significantly enhanced ATP secretion (**Fig 5A**). Correspondingly, CBX and PB completely abolished *Eh*-induced activation of caspase-1 and IL-1β, whereas FFA enhanced the response. Thus, levels of extracellular ATP tune the degree of inflammasome activation (**Fig 5B**). Furthermore, these data suggested that ATP release occurred through a pannexin channel. Of the three pannexins, Panx1 is known to conduct ATP [26]. Incubation of macrophages in a mimetic blocking peptide of Panx1, ^10^Panx1 corresponding to the first extracellular loop of Panx1, but not a scrambled control, abolished ATP release in response to *Eh* and r*Eh*CP5, and prevented activation of caspase-1 and IL-1β [27] (**Fig 5A and 5C**). Similarly, we found that *panx1* ^*-/-*^ macrophages, which had a normal inflammasome response to extracellular application of ATP and is consistent with other reports [28, 29], did not activate the inflammasome in response to *Eh* (**Fig 5D**). Together, these data indicate that Panx1 channels conduct ATP into the extracellular space triggered by activation of α_5_β_1_ integrin by *Eh*CP5.

A recent study demonstrated that caspases 3 and 7 regulate Panx1 opening by cleaving the C-terminus of Panx1 during apoptosis, and *Eh* can activate caspase 3 [30, 31]. To test whether α_5_β_1_ integrin activation lead to caspase-dependent opening of Panx1 we assessed Panx1 for cleavage after *Eh* contact, assuming that the C-terminal directed Panx1 antibody would show either a mobility shift or decrease in band intensity of Panx1, as was previously reported [30]. However, we observed no changes to Panx1 in *Eh*-stimulated macrophage lysates (**Fig 5E**). To address this further we applied the pan-caspase inhibitor zVAD and measured ATP release. We found that zVAD did not inhibit ATP release in response to *Eh* or to r*Eh*CP5, ruling out possibility of this pathway (**Fig 5E**).

Activation of purigenic receptors and NMDA receptors leads to SFK-dependent opening of Panx1, raising the possibility that α_5_β_1_ integrin activates SFKs to directly regulate Panx1 [32, 33]. A conserved SFK phosphorylation site has been identified at Y308 in the C-terminus of mouse Panx1, which corresponds to Y309 of human Panx1 [32]. An interfering C-terminal peptide corresponding to this conserved region has been used to attenuate opening of Panx1 in response to SFK-dependent signaling [32]. To investigate whether SFK phosphorylation of Panx1 at Y309 was involved we applied the small interfering sequence comprising the amino acids 306–319 of human Panx1 fused to a TAT sequence for membrane permeability, which is the same sequence that was used to block opening of mouse Panx1. This region of Panx1 is 100% conserved between mouse and human, which additionally suggests this region of Panx1 is critical for function. Application of the interfering peptide, which we termed Panx1_306-319_, inhibited ATP release in response to *Eh* and to r*Eh*CP5, while the TAT control peptide did not (**Fig 5F**). Furthermore, Panx1_306-319_ but not the Tat control does-dependently inhibited *Eh*-induced activation of caspase-1 and IL-1β (**Fig 5G**). Our data therefore, indicate that activation of α_5_β_1_ integrin upon ligation of *Eh*CP5 leads to SFK phosphorylation of Panx1 and opening of the channel to mediate rapid release of ATP.

### *Eh*CP5 cysteine protease activity is required to activate α_5_β_1_ integrin, ATP release and the NLRP3 inflammasome

**Fig 6:**
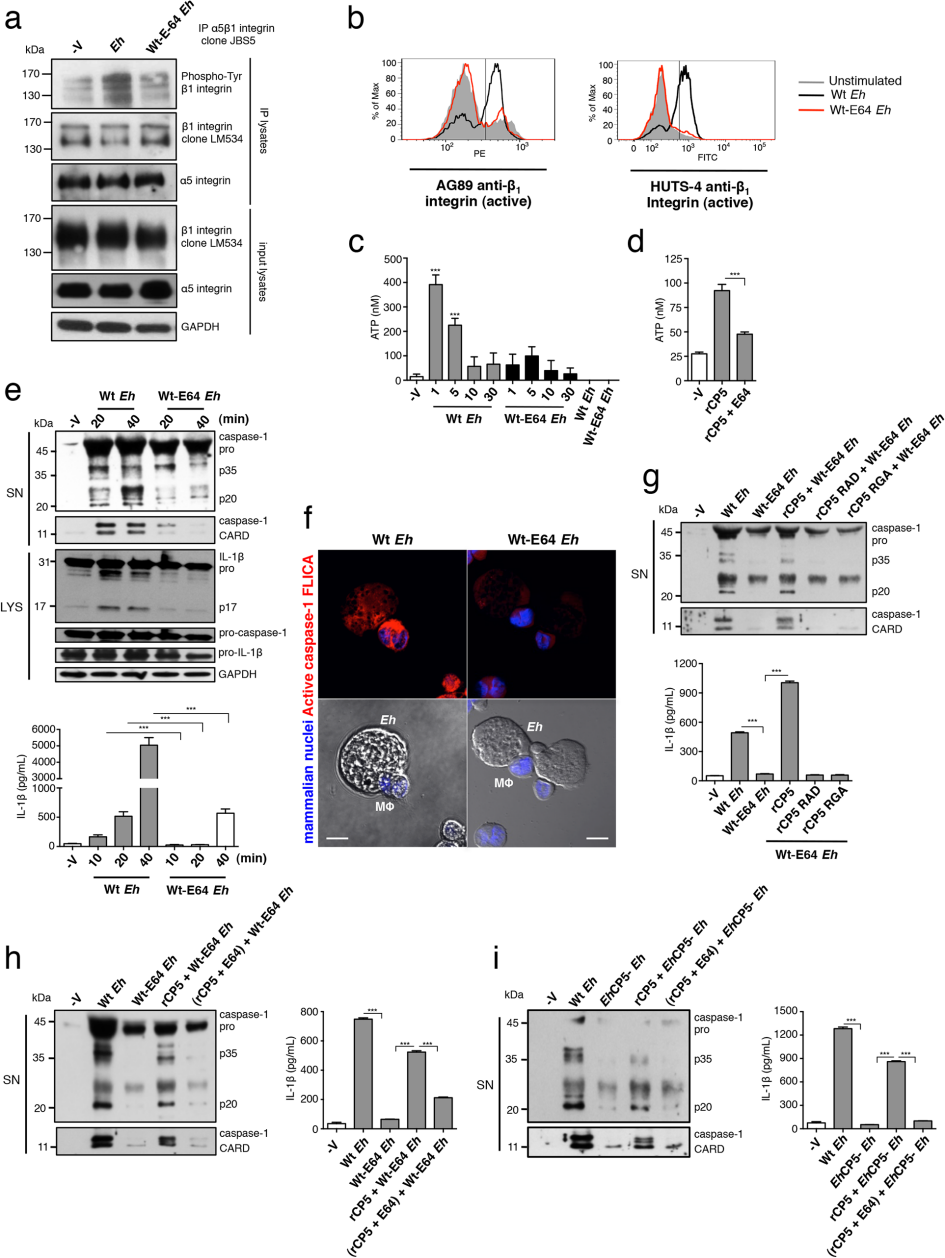
*Eh*CP5 cysteine protease activity is required to activate α_5_β_1_ integrin, ATP release and the NLRP3 inflammasome. (**a**) Immunoblot analysis of β_**1**_ integrin tyrosine phosphorylation (clone PY20) of anti-α_**5**_β_**1**_ integrin immunoprecipitates from PMA-differentiated THP-1 macrophages stimulated with Wt *Eh* or Wt-E64 *Eh*. (**b**) Activation status of β_**1**_ integrin in THP-1 macrophages stimulated with Wt *Eh* or Wt-E64 *Eh* evaluated with anti-β_**1**_ integrin mAbs, AG89 and HUTS-4 that recognize the active conformation specific epitope of β_**1**_ integrin. (**c, d**) ATP release from THP-1 macrophages stimulated for 1 min with a 1:30 ratio of Wt *Eh* or Wt-E64 *Eh*, or rCP5 (1 μg mL^-1^), or rCP5 inactivated first in E-64 (100 μM). (**e, g, h, i**) Immunoblot analysis of IL-1β and caspase-1 activation and IL-1β enzyme-linked immunosorbent assay of PMA-differentiated THP-1 macrophages stimulated for 30 min or indicated time with Wt *Eh* (**e, g, h, i**), Wt-E64 *Eh* (**e, g, h**), *Eh*CP5- *Eh* (**i**) alone or with the addition of rCP5 (1 μg mL^-1^) (**g, h, i**), rCP5 RAD (**g**), rCP5 RGA (**g**) or rCP5 inactivated in E-64 (100 μM) (**h, i**). (**f**) Caspase-1 activation upon contact with Wt or Wt-E64 *Eh* in which active caspase-1 was stained with YVAD-FLICA, red; mammalian nuclei, blue (*Eh* nuclei are not stained). Scale bars, 10 μM. Data are representative of two (**c, f**) or three (**a, b, d, e, g-i**) separate experiments (error bars SEM). ***P < 0.005.

Besides its integrin-binding RGD sequence, the cysteine protease is the major functional domain of *Eh*CP5 [34]. We examined whether the cysteine protease played a role in activating α_5_β_1_ integrin and the pathway leading to NLRP3 activation. Wt *Eh* were grown overnight in presence of the irreversible cysteine protease inhibitor E-64 (hereafter referred to as Wt-E64 *Eh*), remain viable and express enzymatically inactive *Eh*CP5 [35]. We first tested their ability to activate α_5_β_1_ integrin. To our surprise Wt-E64 *Eh* did not induce phosphorylation of α_5_β_1_ integrin or expression of the active β_1_ epitope (**Fig 6A and 6B**). Thus, the cysteine protease was essential to induce activation of α_5_β_1_ integrin, and indicated it would be essential to induce ATP release and inflammasome activation. We found that Wt-E64 *Eh* and r*Eh*CP5 that was first inactivated in E-64 did not induce ATP release (**Fig 6C and 6D**). As predicted by these data, Wt-E64 *Eh* did not activate caspase-1 or IL-1β (**Fig 6E and 6F**). Addition of r*Eh*CP5 to Wt-E64 *Eh* restored inflammasome activation in an RGD-dependent manner (**Fig 6G**) while, addition of r*Eh*CP5 in which cysteine protease activity was abolished did not restore inflammasome activation to Wt-E64 *Eh* or to *Eh*CP5^-^ *Eh* (**Fig 6H and 6I**). Therefore, the cysteine protease and the RGD sequence of *Eh*CP5 are essential to trigger α_5_β_1_ integrin-induced ATP release to activate the NLRP3 inflammasome. Note that α_5_β_1_ integrin does not undergo cleavage upon *Eh* contact and we were not able to identify the proteolytic target of *Eh*CP5 triggering α_5_β_1_ integrin activation. Our data indicated the combined role of *Eh*CP5 RGD sequence and cysteine protease was to induce ATP release that stimulates NLRP3. To determine if this was the singular function of *Eh*CP5, we added exogenous ATP to macrophages stimulated with *Eh*CP5^-^ *Eh* or Wt-E64 *Eh* (this concentration of ATP together with TLR agonists stimulates NLRP3). Unexpectedly, exogenous ATP did not restore inflammasome activation (**S8 Fig**). This indicates that *Eh*CP5 initiates additional signalling that is critical for NLRP3 activation, which might involve α_5_β_1_ integrin or other surface receptors.

### *Eh*CP5 and the NLRP3 inflammasome control intestinal IL-1β responses

**Fig 7:**
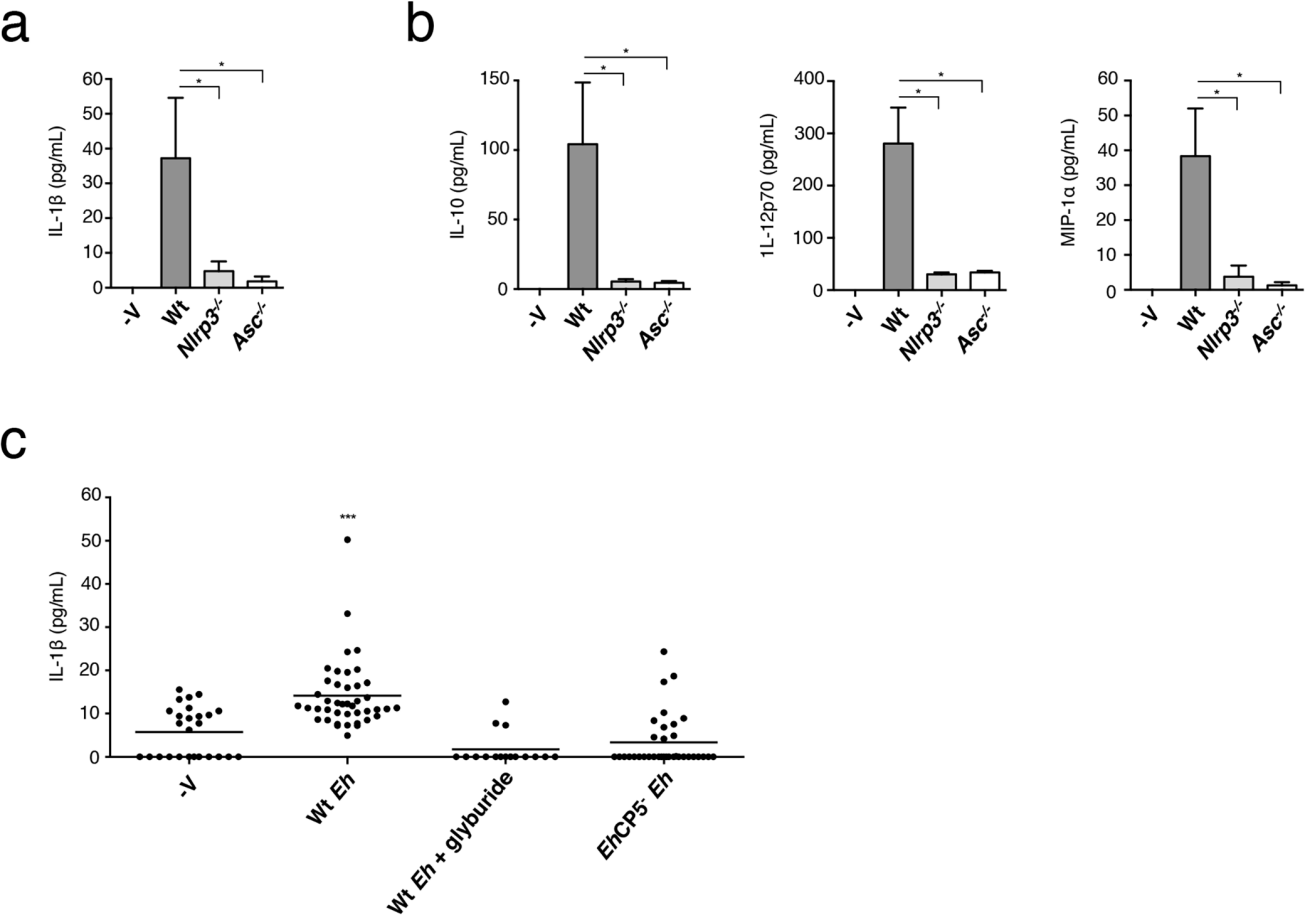
NLRP3 inflammasome is involved in *E*. *histolytica*-induced inflammatory responses of the colon. Cytokine array of IL-1β (**a**), IL-12p70 (**b**), MIP-1α (**b**) and IL-10 (**b**) of luminal secretions from colons challenged with 1 x10^6^ Wt *Eh* for 3 h (n = 4 per group). *P < 0.05 (unpaired Mann-Whitney test). (**c**) Enzyme-linked immunosorbent assay of IL-1β secretion from normal healthy human colonic biopsies stimulated with Wt *Eh* with or without Glyburide (100 μM), or *Eh*CP5^-^ *Eh* for 3 h. Each symbol represents an individual biopsy (control, n = 26; Wt *Eh*, n = 46; Wt *Eh* + Glyburide, n = 16; *Eh*CP5- *Eh*, n = 36) ***P < 0.005 (unpaired student’s *t*-test). Data are representative of one (**a, b**) and three or more pooled experiments (**c**).

During acute *Eh* infection rapid innate immune responses are vital for early detection and swift responses to invasion across the mucosal barrier to restrict and eliminate infectious spread. To define whether NLRP3 inflammasome activation regulates IL-1β secretion during acute *Eh* infection, we compared the early innate response at 3 h in closed proximal colonic loops of mice infected with *Eh*. In this model we observe a rapid secretory response characterized by distension of the colon by intense luminal secretions that are comprised of water, mucus, variable amounts of blood and pro-inflammatory cytokines [36]. This is consistent with the human disease where invasion by *Eh* most often causes diarrhea and dysentery [1]. Gross pathology was similar between Wt and *nlrp3* ^-/-^ animals. *asc* ^-/-^ animals, however, tended towards a more severe response with visible bloody luminal exudates, inflamed dilated blood vessels and a larger secretory response. To determine IL-1β secretion we measured cytokine secretion in luminal exudates of infected animals. While there was IL-1β in luminal exudates of Wt mice, it was virtually undetectable in the luminal secretions of *nlrp3* ^-/-^ and *asc* ^-/-^ mice (**Fig 7A**). Furthermore, other inflammation-related cytokines were absent in *nlrp3* ^-/-^ and *asc* ^-/-^ mice (**Fig 7B**). To address whether IL-1β secretion occurs during invasion in human colonic tissues, we incubated healthy human colonic biopsies for 3 h with *Eh* in the presence or absence of the NLRP3 inhibitor glyburide. Glyburide was not directly toxic to *Eh* and *Eh* treated with glyburide activate the inflammasome. As predicted, glyburide completely abrogated IL-1β secretion (**Fig 7C**). We previously established that *Eh*CP5^-^ *Eh* do not induce IL-1β in the colonic loop model [36]. To test this in human colon we measured IL-1β secretion from human biopsies stimulated with *Eh*CP5^-^ *Eh*. In contrast to Wt *Eh*, *Eh*CP5^-^ *Eh* did not elicit IL-1β from human colon (**Fig 7C**). Taken together, these data show that *Eh*CP5-triggered activation of the NLRP3 inflammasome and regulated IL-1β release during acute invasion of the intestinal mucosa.

## Discussion

The immune mechanisms that escalate the inflammatory response to invasive *Eh* have been amongst the most significant questions of the pathogenesis of amebiasis. We recently identified that attachment of *Eh* to macrophages mediated by *Eh*-Gal-lectin triggers inflammasome activation [8]. Until this point, direct contact of the parasite with cells of the innate immune system had not been recognized as an event that initiates inflammation. This lead us to speculate that engagement of surface receptors and their organization into the intercellular junction critically regulate inflammasome activation and this was a sensing mechanism for distinguishing immediate microbial contact to activate a highly potent inflammatory response. The present gap in knowledge of host receptors that interact with *Eh*-Gal-lectin needs to be addressed to understand how contacts with macrophages form and “adhesive” signaling that may regulate NLRP3 activation. However, because *Eh*-Gal-lectin was a prerequisite for adhesion, we considered if additional molecules on the surface of *Eh* engage surface receptors and contribute to *Eh*-sensing once principal contact has been established by *Eh*-Gal-lectin.

We had previously established that *Eh*CP5, a cysteine protease both secreted and expressed on the surface of *Eh* could ligate α_v_β_3_ integrin on colonic epithelia via an RGD sequence, which activated Akt and led to NF-κB-driven production of pro-inflammatory cytokines [14]. In these studies this interaction was explored using the soluble protein and a fragment of *Eh*CP5 containing the RGD-sequence. However, further sequence analysis revealed that *Eh*CP5 contains an RNS sequence, which in fibronectin, a canonical ligand of α_5_β_1_ integrin, synergizes with the RGD to specify binding to α_5_β_1_ integrin. This indicated that *Eh*CP5 was likely to interact with α_5_β_1_ integrin [16]. Additionally, given the essential role of integrins in cell adhesion and polarization and that key features that regulate their level of activation are the size of contact surfaces and receptor clustering, this raised the possibility that surface-bound *Eh*CP5 could trigger integrin activation in a very different manner to soluble integrin ligation and that integrins could be involved in the ability of host cells to distinguish direct *Eh* contact [15]. This served as the basis to explore the role of integrins in contact-dependent activation of the inflammasome.

Here we found that NLRP3 is the inflammasome activated by direct *Eh* contact with macrophages and intriguingly, we observed recruitment of NLRP3 into the microenvironment of the intercellular junction, indicating that signaling from this location was critical to activate NLRP3. On the cell surface we saw that α_5_β_1_ integrin was strongly recruited and the presence of phosphorylated-paxillin revealed these were locations of active integrin signaling. We confirmed there was direct activation of α_5_β_1_ integrin upon *Eh* contact by showing there was SFK-dependent phosphorylation of the β_1_ subunit in immunoprecipitates of α_5_β_1_ integrin and expression of the active epitope of β_1_ integrin when macrophages were stimulated with *Eh*. As phosphorylation of the β_1_ subunit was inhibited by addition of a soluble RGD peptide, which prevents RGD-binding integrins from interacting with RGD-containing ligands, we investigated whether *Eh*CP5 could ligate and activate α_5_β_1_ integrin. We found that soluble *Eh*CP5 ligated α_5_β_1_ integrin, whereas *Eh*CP5 with a mutated RGD sequence did not and that *Eh*CP5-*Eh* did not induce phosphorylation of the β_1_ subunit or expression of the active β_1_ epitope. These data showed *Eh*CP5 was responsible for activating α_5_β_1_ integrin upon contact. We could not, however, detect α_5_β_1_ integrin activation by these methods with soluble *Eh*CP5, suggesting that α_5_β_1_ integrin could be involved in distinguishing direct *Eh* contact that lead to NLRP3 inflammasome activation. We tested this hypothesis by disrupting RGD interactions and SFK activation, which is critical to initiate integrin signaling, and both abolished inflammasome activation. A panel of integrin function-blocking antibodies against the RGD-binding integrins that are expressed on THP-1 macrophages revealed that only inhibition of α_5_β_1_ integrin could block inflammasome activation, which we corroborated by individually silencing expression of the α_5_ and β_1_ subunits. Thus, our data clearly show that activation of α_5_β_1_ integrin via direct contact with *Eh* was required to activate the NLRP3 inflammasome.

In the immune system high concentrations of extracellular ATP is a danger signal and in macrophages activates the NLRP3 inflammasome in the setting of a pathogen stimulus. It was of interest to us that the rapid inflammasome activation by *Eh* paralleled the rate and magnitude of ATP stimulation. To understand how α_5_β_1_ integrin activation controlled NLRP3 we explored whether ATP functioned in this pathway. Hydrolysis of ATP and interference of P2X_7_R activation established that ATP was indeed involved and we showed that trophozoites and to a lesser degree soluble *Eh*CP5 stimulated a burst of ATP, which required the activation of α_5_β_1_ integrin by *Eh*CP5. We showed *Eh*CP5 could directly trigger release of ATP from macrophages through its interaction with α_5_β_1_ integrin. Though we could not detect enhanced phosphorylation and expression of the active β_1_ epitope by soluble *Eh*CP5, our data indicate that binding of soluble *Eh*CP5 to some extent ‘tickled’ the integrin to stimulate a small amount of ATP release.

ATP can be released through pannexin and connexin channels, which allow the diffusion of ions across the plasma membrane [25]. In immune cells Panx1 appears particularly important for this [25]. We found that pannexin family and Panx1-specific channel blockers abolished ATP release and inflammasome activation by *Eh*. In further support, we tested *Eh*-induced inflammasome activation in *Panx1* ^*-/-*^ macrophages, which in our hands and reported by others activate the inflammasome normally in response to extracellular ATP [27, 28]. However, inflammasome activation did not occur in *Panx1* ^*-/-*^ macrophages stimulated by *Eh*. This fits with Panx-1 channels conducting ATP upon contact, such that when Panx1 is absent ATP is not released and the NLRP3 inflammasome is not activated. Finally to understand how α_5_β_1_ integrin could regulate opening of Panx1 we tested two possibilities. First, whether this was by caspase cleavage of the Panx1 intracellular C-terminus, which has been shown to regulate ATP release in apoptotic immune cells [29]. However, we ruled this pathway out as we never observed a band shift or disappearance of any Panx1 glycoforms after *Eh* stimulation and ATP release was not sensitive to pan-caspase inhibition. We considered whether another pathway involving SFK phosphorylation of a conserved region of the Panx1 intracellular C-terminus regulated ATP secretion. Though phosphorylation of Panx1 by SFKs has not been demonstrated directly, two groups have shown that SFKs can open Panx1 and an SFK consensus sequence has been identified in Y308 of mouse Panx1 [32, 33]. The region surrounding this sequence is completely conserved in human Panx1 and indicates this region is critical for Panx1 function. Using the same strategy employed by these groups, we showed that PP1 prevented ATP release and an interfering C-terminal peptide comprising the amino acids 309–319 of the Panx1 C-terminus, which covers the predicted SFK phosphorylation sequence, blocked ATP release and inhibited activation of the NLRP3 inflammasome by *Eh*. Together our data suggest when α_5_β_1_ integrin is activated via direct contact with *Eh*, SFKs that become instantly active upon integrin activation, are involved in phosphorylating the Panx1 intracellular domain causing the channel to open and release ATP into the extracellular space.

*Eh* invasion initiates vigorous tissue inflammation. *Eh*CP5^-^ *Eh* are avriulent in models of *Eh* infection and elicit little inflammation indicating *Eh*CP5 is essential for triggering inflammatory responses [15, 36–38]. Interestingly, an early report suggested if pro-IL-1β was released, *Eh* cysteine proteases could cleave IL-1β into the active form [39]. Our *in vivo* results in an animal model of acute *Eh* invasion and in intact human colonic biopsies cultured with the parasite indicate the NLRP3 inflammasome plays a significant role in regulating the pro-inflammatory response in acute intestinal amebiasis by regulating IL-1β responses in the colon.

In immune cells, signaling at intercellular junctions involving large-scale reorganization of surface receptors and intracellular signaling molecules, collectively termed ‘immune synapses,’ have long been recognized to control the activation of adaptive immune cells [40]. Recently, this concept was extended to microbial recognition by macrophages in deciphering particulate versus soluble ligands that bind to the same receptors but initiate different responses [41]. In this work the authors showed particulate fungal β glucan is distinguished from soluble β glucan as a result of Dectin-1 clustering and spatial exclusion of the phosphatase CD45, which triggered signaling that lead to phagocytosis and release of reactive oxygen, and is proposed as a mechanism to distinguish and activate direct anti-fungal responses only when fungi are immediately present [41]. Our data demonstrate that NLRP3 and α_5_β_1_ integrin cooperate to distinguish direct contact with extracellular microbes where NLRP3 is recruited into the synapse and undergoes activation via ATP signalling as a consequence of α_5_β_1_ integrin activation in the intercellular junction. In this way inflammasome-induced pro-inflammatory responses are restricted and localized to locations where *Eh* are present. This is reminiscent of other immune synapses in which long-range recruitment of molecules into the junction critically regulates their activity and cell activation. Contact-dependent release of ATP through opening of Panx1 was a critical part of the mechanism through which α_5_β_1_ integrin regulated NLRP3. Similarly, activation of T cell immune synapses requires a large burst of ATP through Panx1 [42, 43]. In this regard, the magnitude of ATP release that occurred following macrophage-*Eh* contact is similar to cross-linking the CD3 receptor on T cells and thus contact-dependent triggering of ATP may be another common feature regulating immune cell synapses.

A recent study revealed α_5_β_1_ integrin is involved in NLRP3 inflammasome activation by a soluble toxin Td92 from the periodontal pathogen *Treponema denticola* [44]. However, though soluble Td92 directly ligated α_5_β_1_ integrin through a non-RGD interaction, the mechanism through which NLRP3 was regulated by α_5_β_1_ integrin appears to be different to its role in sensing *Eh* contact. Td92 activated the inflammasome several hours after stimulation and required α_5_β_1_ integrin to activate NF-κB, which was essential not only for pro-IL-1β induction but also for caspase-1 activation [44]. Thus, α_5_β_1_ integrin likely regulated NLRP3 indirectly through a transcription-dependent event. In sensing *Eh* contact, however, α_5_β_1_ integrin engages post-translational signalling that rapidly triggers NLRP3. Taken together, this study and ours indicate α_5_β_1_ integrin is a critical receptor for detecting extracellular pathogens linked to the NLRP3 inflammasome. As α_5_β_1_ integrin is commonly exploited by microbial pathogens our data suggest it might be under immune-surveillance by NLRP3 for abnormal activity that would indicate pathogen presence.

In summary we have identified α_5_β_1_ integrin is a critical surface receptor in the intercellular junction that mediates rapid activation of the NLRP3 inflammasome, enabling the host to distinguish direct contact with extracellular *Eh* and mobilize highly pro-inflammatory host defenses precisely at sites of *Eh* infection.

## Materials and Methods

### Reagents

Ultra-pure LPS, PMA, apyrase, DPI, KCL, ATP, oATP, KN-62, cytochalasin D, bafilomycin, glyburide, E-64 were from Sigma. zVAD-fmk, YVAD-fmk were from Enzo Life Sciences. GRGDSP, GRADSP, PP1, PP3 were from Calbiochem. ^10^Panx1 (WRQAAFVDSY) and ^10^Panx1-scrambled peptides were from Anaspec. Integrin function blocking antibodies were from Millipore. MSU was a gift from Y. Shi (University of Calgary). Panx1^309^ and Tat peptides were a gift from R. Thompson (University of Calgary). Human and murine IL-1β secretion in cell culture was quantified by enzyme linked immunosorbent assay (R&D Systems).

### Animals

C57BL/6 mice were from Charles River. The following genetically modified mouse strains were used all on a C57BL/6 background: *nlrp3* ^*-/-*^ (D. Muruve and P. Beck, University of Calgary), *asc* ^*-/-*^ (D. Muruve and P. Beck, University of Calgary), *panx1* ^*-/-*^ (E. Lazarowski, University of North Carolina) and compared to Wt littermates. The University of Calgary Animal Care Committee approved experiments involving animals.

### Cell preparation and stimulation

THP-1 human monocytic cells (ATCC) were plated onto 24 well plates at 4x10^5^ cells/well with 50 ng/mL PMA in complete RPMI 1640 the night before treatment. Cells were stimulated with *Eh* or *rEh*CP5 proteins in 250 μL serum-free RPMI for the indicated times (1–40 min). For inhibitor studies THP-1 cells were pre-treated at 37°C unless otherwise stated with the indicated concentrations of zVAD-fmk (100 μM, 45 min), YVAD-fmk (100 μM, 45 min), DPI (25–100 μM, 30 min), KCL (90–200 mM was added to fresh serum-free media upon *Eh*), glyburide (100 μM, 30 min), cytochalasin D (5–10 μM, 30 min), bafilomycin (50–250 nM, 30 min), oATP (50–100 μM, 2 h), KN-62 (1–5 μM, 30 min), apyrase (20 U mL^-1^, 5 min), ^10^Panx1 (500 μM, 10 min RT), ^10^Panx1 scrambled (500 μM, 10 min RT), GRGDSP (50 μM, 10 min RT), GRADSP (50 μM, 10 min RT), integrin function-blocking antibodies (10 μg mL^-1^,10 min RT), PP1 (5–20 μM, 10 min, 1-(1,1-Dimethylethyl)-1-(4-methylphenyl)-1H-pyrazolo[3,4-d]pyrimidin-4-amine) and PP3 (10–20 μM, 10 min, 1-Phenyl-1H-pyrazolo[3,4-d]pyrimidin-4-amine), Panx1^309^ (5–10 μM, 10 min RT), Tat (5–10 μM, 10 min RT) and changed into fresh serum-free media before stimulation with *Eh* unless otherwise stated. BMDM were prepared from the femurs and tibias of mice cultured for 6 days in 10% FBS-RPMI 1640 supplemented with 30% L-cell supernatant, then replated onto 24 well plates at 5x10^5^ cells/well in complete RPMI 1640. On the day of experiment BMDM were treated with 1 μg/mL LPS for 3.5 h. Cells were stimulated with 1.5x10^4^ *Eh* per well in 250 μL serum-free RPMI for the indicated time. As a control for the silencing technology in *Eh*CP5^-^ *Eh* we tested a vector control strain that has the same silencing technology as *Eh*CP5^-^ *Eh*. The *Eh*CP5^-^ *Eh* are also silenced for *Eh*APA gene. As a control for off-target effects and for contribution of *Eh*APA, we tested *Eh* that are only silenced for *Eh*APA, designated *Eh*APA^-^ *Eh*, and these amoeba stimulated inflammasome activation similar to wildtype *Eh*. We concluded neither the silencing technology nor *Eh*APA altered the ability of *Eh* to stimulate inflammasome activation.

### *E*. *histolytica* culture

*E*. *histolytica* HM-1:IMSS were grown axenically in TYI-S-3 medium with 100 U mL^-1^ penicillin and 100 μg mL^-1^ streptomycin sulfate at 37°C in sealed 15 mL borosilicate glass tubes as described previously [45]. To maintain virulence, trophozoites were regularly passed through gerbil livers as described [46]. Ameba were harvested after 72 h of growth by centrifugation at 200 x *g* for 5 min at 4°C and resuspended in serum-free RPMI. *E*. *histolytica* cultures deficient in *EhCP5* and *EhAPA* (vector control) were a gift from D. Mirelman (Weizmann Institute of Science). To irreversibly inhibit *Eh* cysteine protease activity *Eh* were cultured overnight in E-64 (100 μM) as previously described [15].

### siRNA and transfection

Control and siRNA for the indicated genes were from Dharmacon (OnTarget plus SMART pool siRNA). THP-1 cells were transfected with siRNA (100 nM) for 48 h using DOTAP transfection reagent (Roche) according to the manufacturer’s instructions, followed by overnight PMA stimulation.

### Generation and mutation of recombinant *Eh* Cysteine Protease 5

Expression plasmid pJC45 encoding the pro-form of *Eh*CP5 (amino acids 14–317) was a gift from I. Bruchhaus (Bernhard Nocht Institute for Tropical Medicine, Hamburg, Germany). The RGD site of recombinant *Eh*CP5 was mutated to RAD or RGA by QuikChange site-directed mutagenesis kit (Agilent Technologies). Recombinant plasmids were expressed in *Escherichia coli* strain BL21(DE3) [pAPlacIQ]. Recombinant proteins were expressed as insoluble histidine-tagged pro-enzymes and were solubilized, purified and refolded as previously described [34]. Protein purity was >95% as revealed by SDS-PAGE. To confirm that recombinant enzymes were active and mutation of the RGD motif did not alter proteolytic activity of recombinant *Eh*CP5, cysteine protease activity was measured by cleavage of the fluorogenic cathepsin B substrate Z-Arg-Arg-AMC.

### Immunoblot and immunoprecipitation

THP-1 or BMDM supernatants from 4 wells were pooled and centrifuged at 4°C for 5 min at 2000g. Pelleted debris was discarded and supernatants were concentrated by TCA precipitation. For Western blotting, precipitated supernatants were resuspended in 50 μL Laemmli buffer, boiled for 5 min and equal volumes were resolved on 12.5% polyacrylamide gels and transferred to nitrocellulose. For cell lysates, plates were washed in cold PBS before lysis buffer (NaCL, Tris (pH 8), SDS, Triton 100-X, EDTA, PMSF, E-64, leupeptin, aprotonin, supplemented with protease inhibitor cocktail (Sigma)) and centrifuged at 4°C for 15 min at 14000g. For detection of phospho-proteins lysis buffer included NaF, Na_3_VO_4_. Equal amounts of proteins boiled for 5 min in Laemmli buffer were resolved on 7.5–12.5% polyacrylamide gels and transferred to nitrocellulose. Membranes were blocked in 5% skim milk or 3% BSA for phospho-proteins, incubated overnight at 4°C in primary Abs and visualized with secondary HRP-conjugated Abs. Supernatents were detected with SuperSignal Chemiluminescence Reagents (Pierce) and lysates with ChemiLucent ECL detection (EMD Millipore). Primary Abs were anti-IL-1β cleaved human (1:1000, 2021, Cell Signaling), anti-IL-1β human (1:700, 7884, Santa Cruz), anti-caspase-1 human (1:1000, 2225, Cell Signaling), anti-caspase-1 human (1:1000, 622, Santa Cruz), anti-IL-1β mouse (1:1000, AF401, R&D Systems), anti-caspase-1 mouse (1:1000, 514, Santa Cruz), anti-NLRP3 (1:1000, AG-20B-0014, Adipogen), anti-ASC (1:1000, 30153, Santa Cruz), anti-phosphotyrosine (1:1000, clone PY20, BD Biosciences), anti-β_1_ integrin (1:2000, 1981, Millipore), anti-α_4_ integrin (1:1000, 14008, Santa Cruz), anti-*Eh*CP5 (1:1000, gift from S. Reed, University of San Diego), anti-GAPDH (1:10000, Jackson Laboratories). Antibodies against α_5_β_1_ integrin (Millipore, 1965) were used to precipitate proteins from cell lysates in the presence of 20 μL protein A/G beads (Santa Cruz) overnight at 4°C. Protein complexes were washed three times with lysis buffer and incubated at 95°C for 5 min and resolved by immunoblot analysis.

### Confocal microscopy

For imaging of active caspase-1 THP-1 cells were seeded on glass coverslips and stimulated with *Eh* or LPS (25 ng mL^-1^) and nigericin (5 μM). Cells were washed in cold PBS and fixed in cold acetone for 5 min. YVAD-FLICA (FLICA 660-Caspase-1 reagent, Immunochemistry Technologies) at 1:70 dilutions was added for 1 h at room temperature. Cells were washed twice in PBS-Tween (0.1%) for 5 min and mammalian nuclei were stained with DAPI for 20 min, followed by 2 washes in PBS-Tween (0.1%) for 5 min. Cells were mounted onto slides and imaged immediately as YVAD-FLICA staining dissipates quickly after fixation. For imaging, α_5_β_1_ integrin, phosphorylated-paxillin and NLRP3 THP-1 cells were seeded on glass coverslips and stimulated with *Eh*. Cells were fixed in 4% PFA for 20 min at RT and stained with antibodies against α_5_β_1_ integrin (Millipore, 1965, 1:250), phospho-paxillin (Tyr118) (Millipore, 07733, 1:250) or NLRP3 (Adipogen, AG-20B-0014, 1:200). Mammalian nuclei were stained with DAPI, F-actin was stained with Alexa fluor 647-conjugated phalloidin and in some instances *Eh* were labeled with CFSE. Cells were analyzed on an Olympus IX8-1 FV1000 Laser Scanning Confocal microscope with a 60x objective.

### ATP measurement

The amount of ATP in the media was quantified using CellTiter-Glo Luminescent Cell Viability Assay (Promega) following the manufacturer’s instructions.

### Flow cytometry

For flow cytometry-based monitoring of β_1_ integrin activation *Eh* were added to cells for the indicated time at 37°C. Cells were then washed with cold PBS and fixed in paraformaldehyde followed by addition of 2 different mAbs antibodies AG89 or HUTS-4, which recognize the active β_1_ integrin conformation-specific epitope and evaluated by flow cytometric analysis.

### Ethics statement

The Health Sciences Animal Care Committee from the University of Calgary, have examined the animal care and treatment protocol (M08123) and approved the experimental procedures proposed and certifies with the applicant that the care and treatment of animals used will be in accordance with the principles outlined in the most recent policies on the “Guide to the Care and Use of Experimental Animals” by The Canadian Council on Animal Care. Informed written consent was obtained from all adult patients. The Calgary Health Research Ethic Board at the University of Calgary approved this study.

### Colonic biopsy studies

Fresh healthy colonic biopsies were obtained from patients undergoing routine screening for colon cancer. None of the patients were taking NSAIDs, aspirin or other anti-inflammatory medication within 7 days of the colonoscopy. Tissue were exposed to 5x10^5^ Wt or *Eh*CP5^-^ *Eh* for 3 h in serum-free Opti-MEM (Invitrogen). IL-1β release into the supernatants was quantified by enzyme-linked immunosorbent assay.

### Colonic loops

Mice (*nlrp3* ^*-/-*^, *asc* ^*-/-*^ and C57BL/6 Wt littermates) were bred in a conventional facility and fasted 8 h before surgery. To generate a colonic loop, mice were anesthetized with ketamine/xylazine and ligatures were generated 1 cm and 3 cm distal of the ileal-cecal junction. Colonic loops were injected with 1x10^6^ log-phase virulent *Eh*. Sham challenged animals were injected with 200 μL of the vehicle PBS. Animals were kept on a 37°C heated blanket for 3 h. IL-1β, IL-12p70, MIP-1α and IL-10 were measured in the colonic loop exudates by multiplex cytokine array (Eve Technologies). The University of Calgary Animal Care Committee approved protocols for experiments involving animals.

### Statistics

All experiments shown are representative of three independent experiments unless otherwise indicated. GraphPad Prism4 was used for statistical analysis. Treatment groups were compared using the paired Student’s *t*-test. Statistical significance was assumed at P< 0.05. Results are displayed as mean +/- standard error of the mean (SE). Colonic loop model was compared using the unpaired Mann-Whitney.

## Supporting Information

S1 Fig Pharmacological inhibition of NLRP3 inhibits inflammasome activation by *E*. *histolytica*. PMA-differentiated THP-1 macrophages treated for 30 min with *Eh* at a 1:30 ratio (**a-c**) with the addition of KCL (**a**), diphenyliodium chloride (**b**) or Glyburide (100 μM) (**c**). Secretion and processing of IL-1β and active caspase-1 cleavage products was determined by immunoblot and enzyme-linked immunosorbent assay. KCL, potassium chloride; DPI, diphenyliodium chloride. Data are representative of three separate experiments (error bars SEM). ***P < 0.005.
(TIF)

S2 Fig Knockdown of NLRP3 and ASC in human macrophages inhibits inflammasome activation by *E*. *histolytica* (*Eh*). THP-1 cells were transfected with siRNA to NLRP3 (**a**) and ASC (**b**), or control siRNA (CTL) for 48 h followed by overnight PMA stimulation. Immunoblot anlaysis of inflammasome molecules and enzyme-linked immunosorbent assay of IL-1β after 30 min stimulation with *Eh* at a 1:30 ratio (**a, b**). Data are representative of three separate experiments (error bars SEM). ***P < 0.005.
(TIF)

S3 Fig Inhibition of tubulin polymerization does not suppress inflammasome activation by *E*. *histolytica* (*Eh*). Immunoblot analysis of secreted active caspase-1 cleavage products and IL-1β enzyme-linked immunosorbent assay of PMA-differentiated THP-1 macrophages treated for 30 min with *Eh* at a 1:30 ratio (**a**) or LPS Nigericin (LN) (**b**) with the addition of colchicine to cultures 30 minute before stimulation. Data are representative of three separate experiments (error bars SEM). ***P < 0.005.
(TIF)

S4 Fig Active α_5_β_1_ integrin is recruited into sites of *E*. *histolytica*-macrophage contact. Subcellular localization of α_5_β_1_ integrin (green) and phosphorylated-(active) paxillin (red) in which PMA-differentiated THP-1 macrophages were left unstimulated or stimulated with *Eh*; mammalian nuclei, blue (*Eh* nuclei are not stained). Scale bars, 10 μM. Data are representative of 2 separate experiments.
(TIF)

S5 Fig *E*. *histolytica* (*Eh*) cysteine protease 5 binds α_5_β_1_ integrin. Co-immunoprecpitation assay of rCP5 or rCP5 RAD and α_5_β_1_ integrin. Immunoblot analysis of anti-α_5_β_1_ integrin immunoprecipitates.
(TIF)

S6 Fig Inflammasome activation by *E*. *histolytica* (*Eh*) requires α_5_β_1_ integrin. Immunoblot analysis of secreted active caspase-1 (**a-c**) and IL-1β (**b**) cleavage products and IL-1β enzyme-linked immunosorbent assay (**a-c**) of PMA-differentiated THP-1 macrophages with the addition of integrin function blocking antibodies (10 μg mL^-1^) to α_v_β_3_ integrin (**a**), β_1_ integrin (**b**), α_4_ integrin (**c**), α_5_ integrin (**c**) to cultures 10 min before stimulation with *Eh* for 20 min. Data are representative of two (**a**) or three (**b, c**) separate experiments (error bars SEM). ***P < 0.005.
(TIF)

S7 Fig Inflammasome activation requires *E*. *histolytica* (*Eh*) cysteine protease 5. **(a)** Immunoblot analysis of secreted active IL-1β (p17) and active caspase-1 cleavage products (p10) and IL-1β enzyme-linked immunosorbent assay of BMDM stimulated for 30 min with Wt *Eh* or *Eh*CP5- *Eh*. Data are representative of three separate experiments. **(b)** Inflammasome activation by the *Eh*CP5- vector control. Immunoblot analysis of secreted active caspase-1 cleavage products and IL-1β enzyme-linked immunosorbent assay of PMA-differentiated THP-1 macrophages stimulated with Wt or the *Eh*CP5- vector control, termed *Eh*APA deficient (*Eh*APA^-^) *Eh*. Data are representative of three separate experiments (error bars SEM).
(TIF)

S8 Fig Exogenous ATP does not restore inflammasome activation in response to *Eh*CP5^-^ *Eh* or Wt-E64 *Eh*. **(a, b)** Immunoblot analysis of secreted active caspase-1 cleavage products and IL-1β enzyme-linked immunosorbent assay of PMA-differentiated THP-1 macrophages stimulated for 30 min with Wt *Eh* or *Eh*CP5- *Eh* **(a)** or Wt-E64 *Eh* **(b)** with or without the addition of ATP (5mM). Data are representative of three separate experiments (error bars SEM).
(TIF)
