# Supplementary figures and images for: The NLRP3 Inflammasome Is a Pathogen Sensor for Invasive Entamoeba histolytica via Activation of α5β1 Integrin at the Macrophage-Amebae Intercellular Junction
Source: PLoS Pathog. 2015 May 8;11(5):e1004887. doi: 10.1371/journal.ppat.1004887 (PMC4425650; doi:10.1371/journal.ppat.1004887)

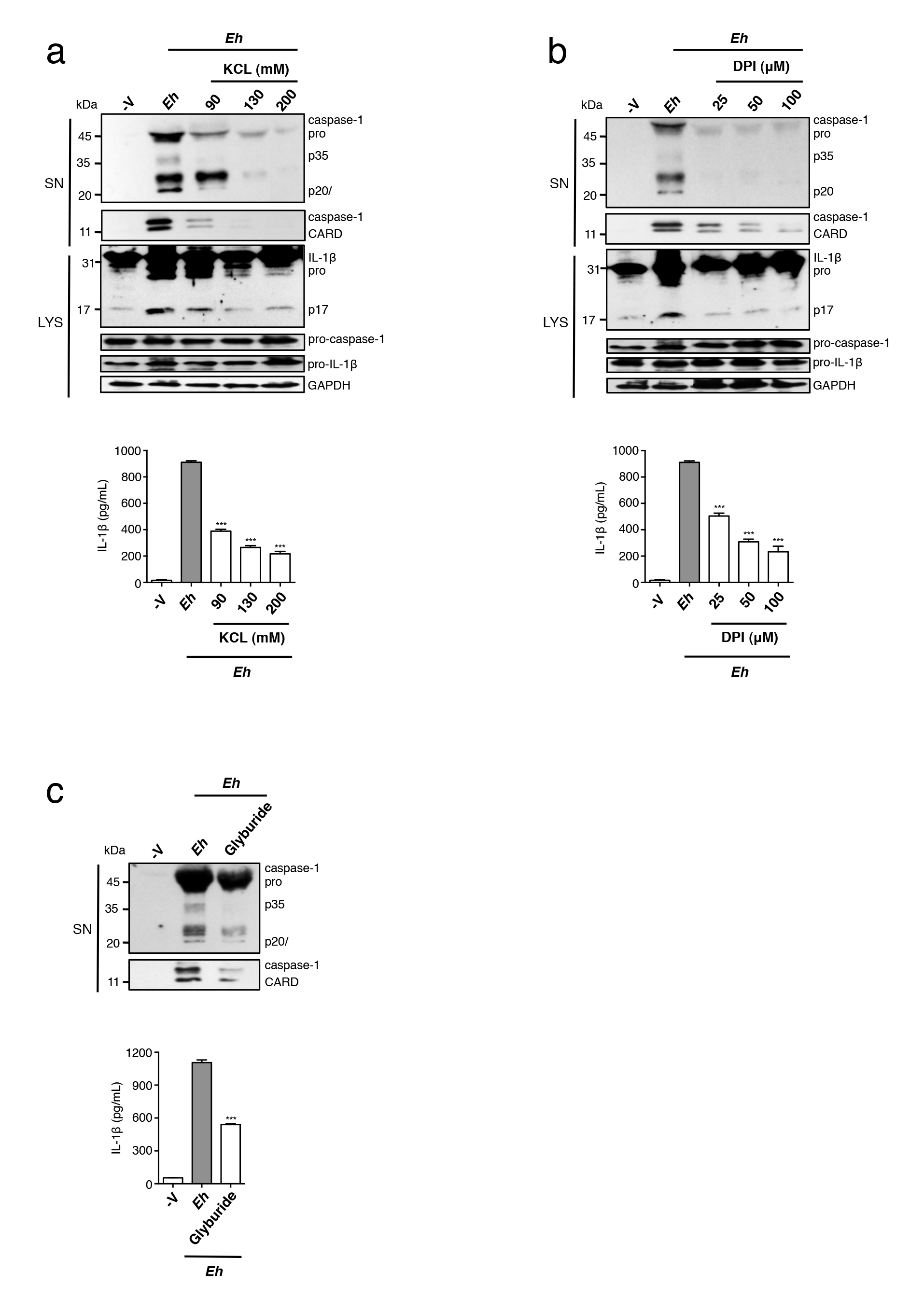

Supplement: S1 Fig — PMA-differentiated THP-1 macrophages treated for 30 min with Eh at a 1:30 ratio (a-c) with the addition of KCL (a), diphenyliodium chloride (b) or Glyburide (100 μM) (c). Secretion and processing of IL-1β and active caspase-1 cleavage products was determined by immunoblot and enzyme-linked immunosorbent assay. KCL, potassium chloride; DPI, diphenyliodium chloride. Data are representative of three separate experiments (error bars SEM). ***P < 0.005. (TIF) [file ppat.1004887.s001.tif]

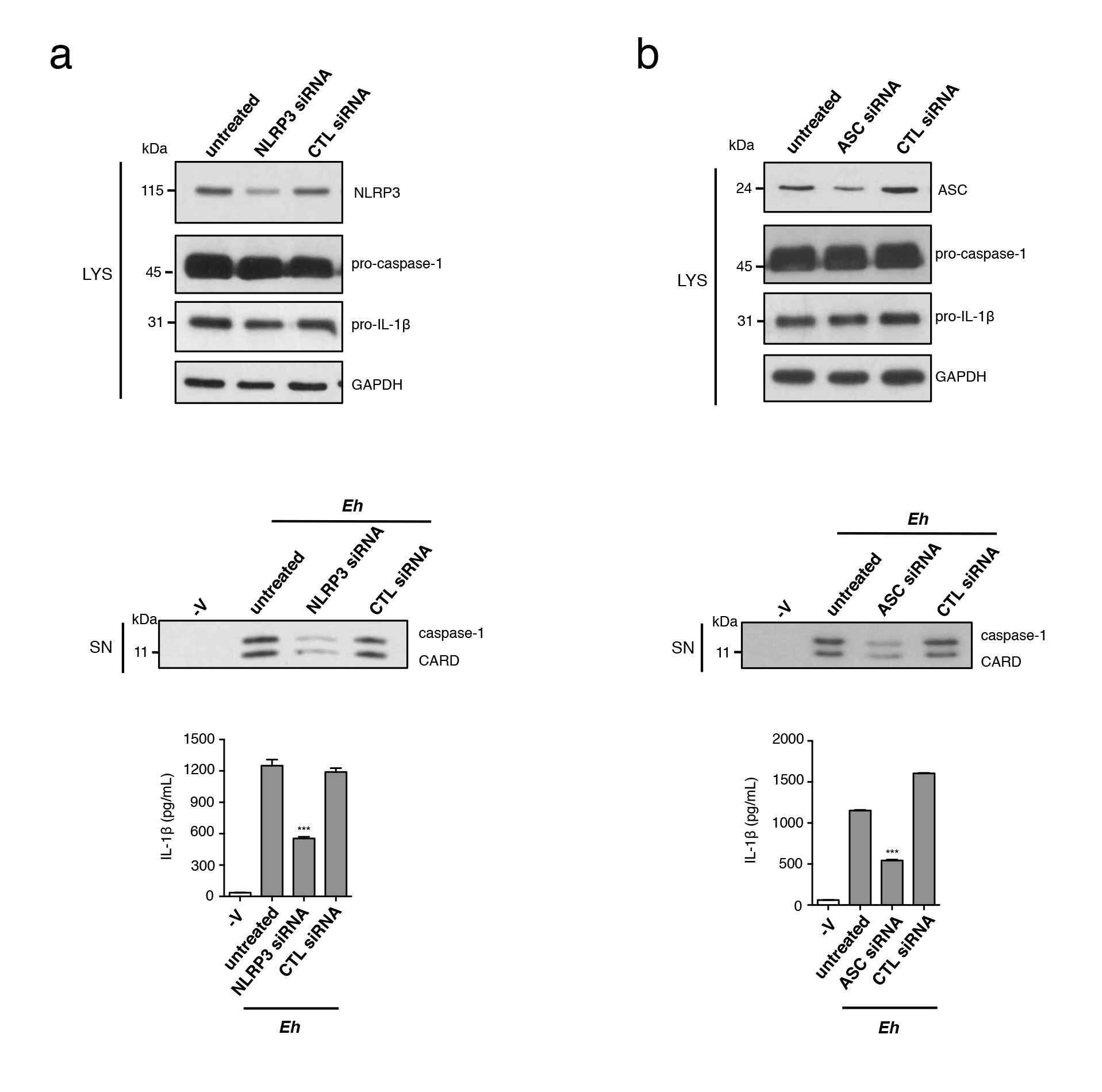

Supplement: S2 Fig — THP-1 cells were transfected with siRNA to NLRP3 (a) and ASC (b), or control siRNA (CTL) for 48 h followed by overnight PMA stimulation. Immunoblot anlaysis of inflammasome molecules and enzyme-linked immunosorbent assay of IL-1β after 30 min stimulation with Eh at a 1:30 ratio (a, b). Data are representative of three separate experiments (error bars SEM). ***P < 0.005. (TIF) [file ppat.1004887.s002.tif]

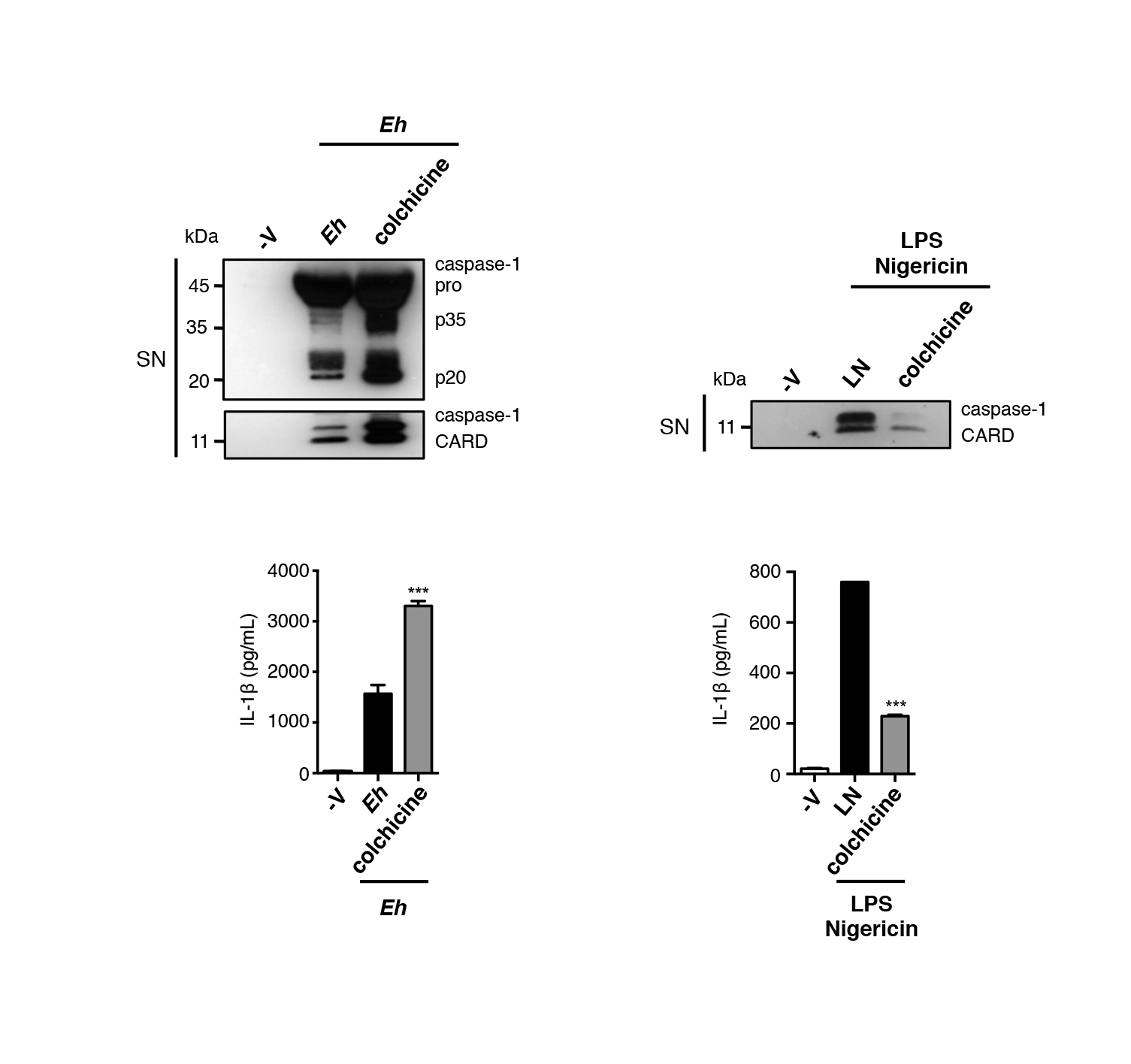

Supplement: S3 Fig — Immunoblot analysis of secreted active caspase-1 cleavage products and IL-1β enzyme-linked immunosorbent assay of PMA-differentiated THP-1 macrophages treated for 30 min with Eh at a 1:30 ratio (a) or LPS Nigericin (LN) (b) with the addition of colchicine to cultures 30 minute before stimulation. Data are representative of three separate experiments (error bars SEM). ***P < 0.005. (TIF) [file ppat.1004887.s003.tif]

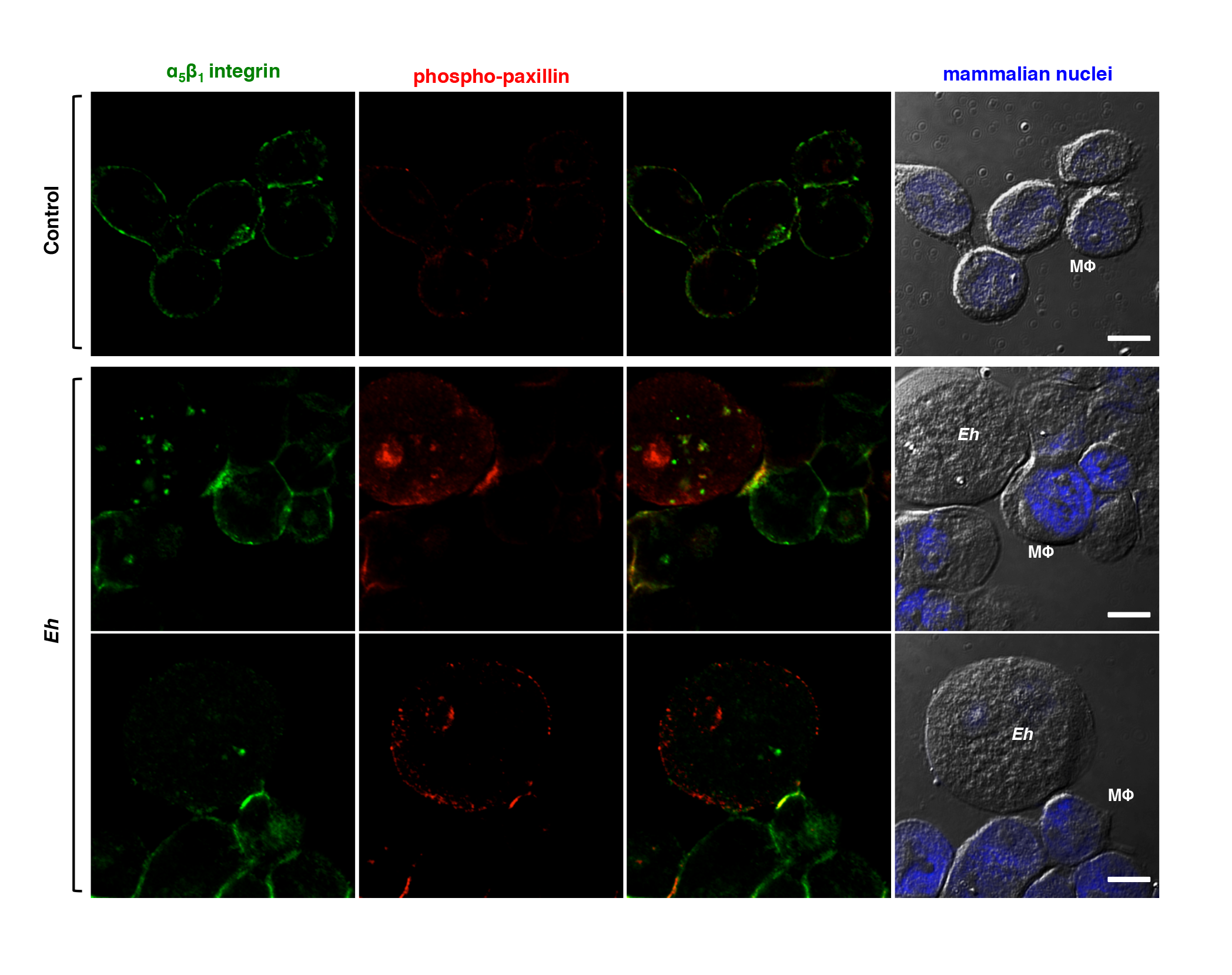

Supplement: S4 Fig — Subcellular localization of α5β1 integrin (green) and phosphorylated-(active) paxillin (red) in which PMA-differentiated THP-1 macrophages were left unstimulated or stimulated with Eh; mammalian nuclei, blue (Eh nuclei are not stained). Scale bars, 10 μM. Data are representative of 2 separate experiments. (TIF) [file ppat.1004887.s004.tif]

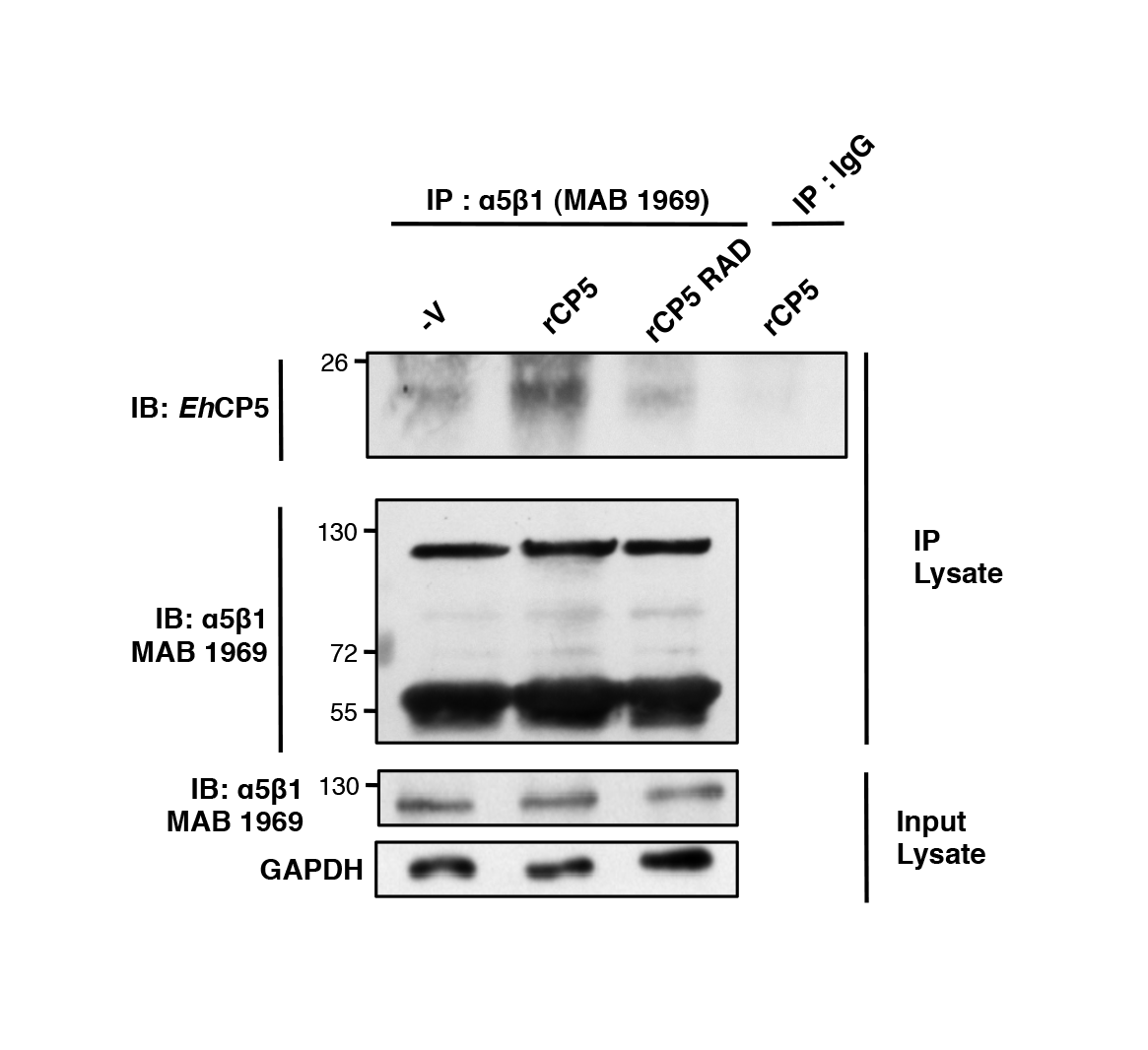

Supplement: S5 Fig — Co-immunoprecpitation assay of rCP5 or rCP5 RAD and α5β1 integrin. Immunoblot analysis of anti-α5β1 integrin immunoprecipitates. (TIF) [file ppat.1004887.s005.tif]

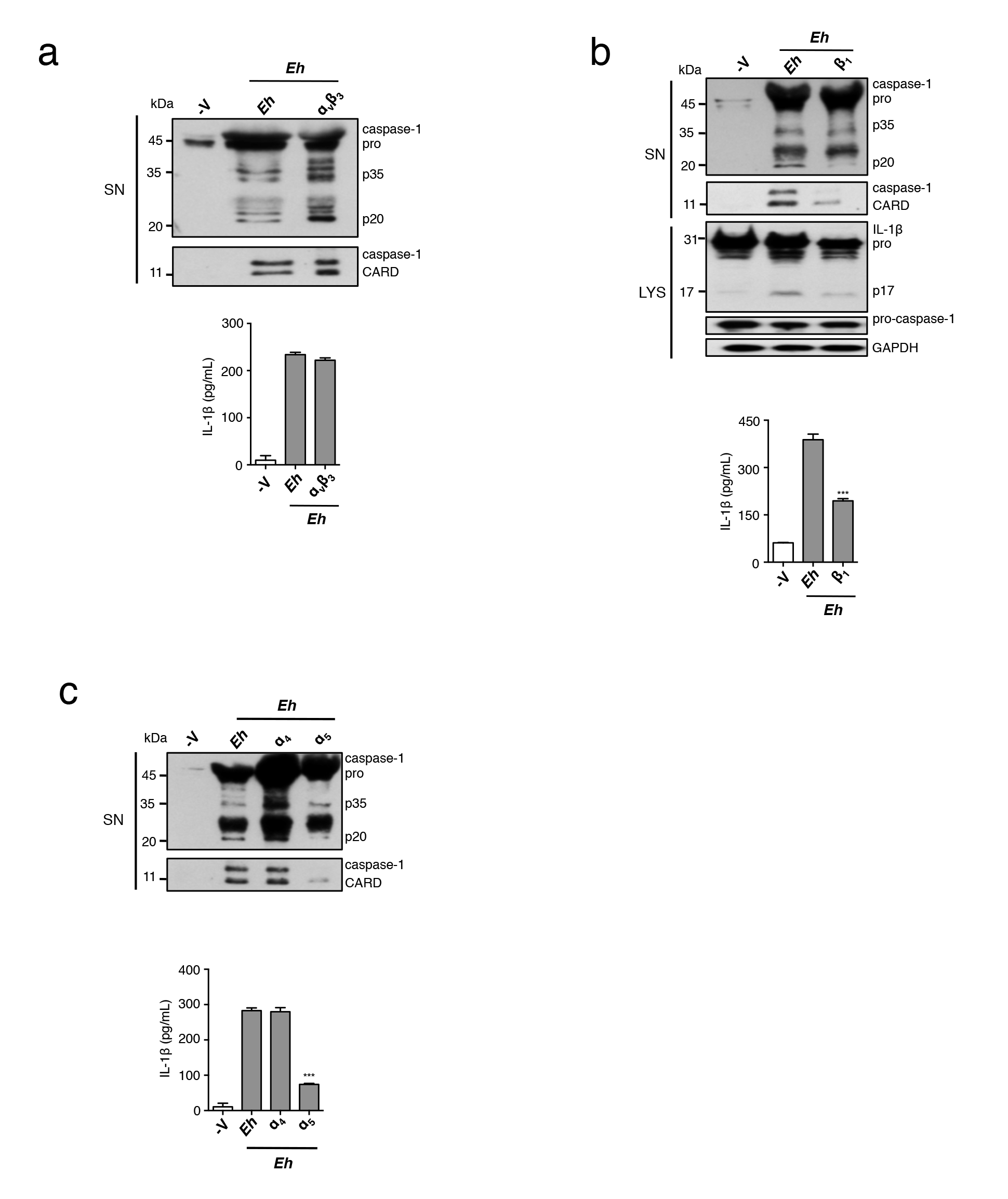

Supplement: S6 Fig — Immunoblot analysis of secreted active caspase-1 (a-c) and IL-1β (b) cleavage products and IL-1β enzyme-linked immunosorbent assay (a-c) of PMA-differentiated THP-1 macrophages with the addition of integrin function blocking antibodies (10 μg mL-1) to αvβ3 integrin (a), β1 integrin (b), α4 integrin (c), α5 integrin (c) to cultures 10 min before stimulation with Eh for 20 min. Data are representative of two (a) or three (b, c) separate experiments (error bars SEM). ***P < 0.005. (TIF) [file ppat.1004887.s006.tif]

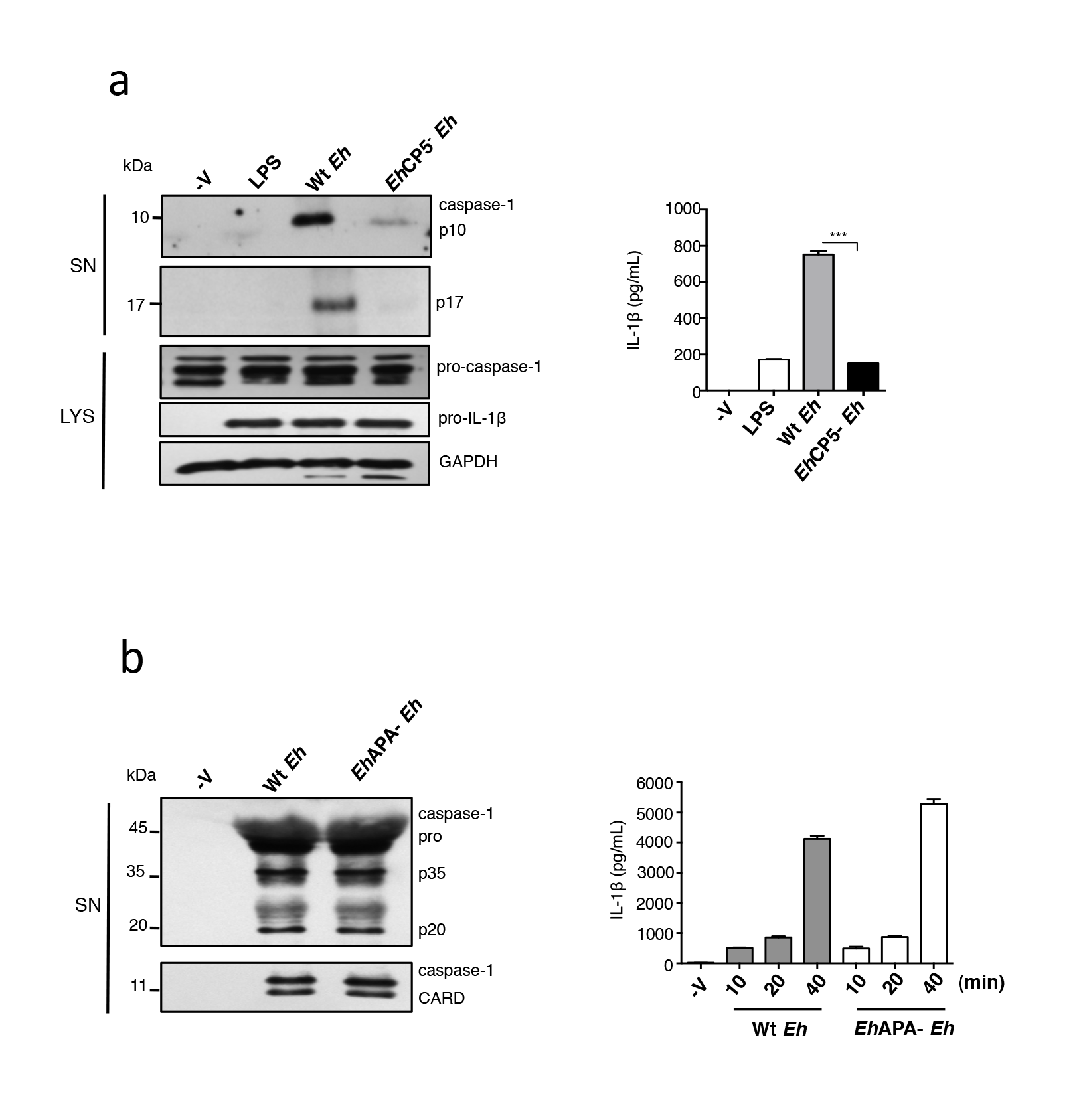

Supplement: S7 Fig — (a) Immunoblot analysis of secreted active IL-1β (p17) and active caspase-1 cleavage products (p10) and IL-1β enzyme-linked immunosorbent assay of BMDM stimulated for 30 min with Wt Eh or EhCP5- Eh. Data are representative of three separate experiments. (b) Inflammasome activation by the EhCP5- vector control. Immunoblot analysis of secreted active caspase-1 cleavage products and IL-1β enzyme-linked immunosorbent assay of PMA-differentiated THP-1 macrophages stimulated with Wt or the EhCP5- vector control, termed EhAPA deficient (EhAPA-) Eh. Data are representative of three separate experiments (error bars SEM). (TIF) [file ppat.1004887.s007.tif]

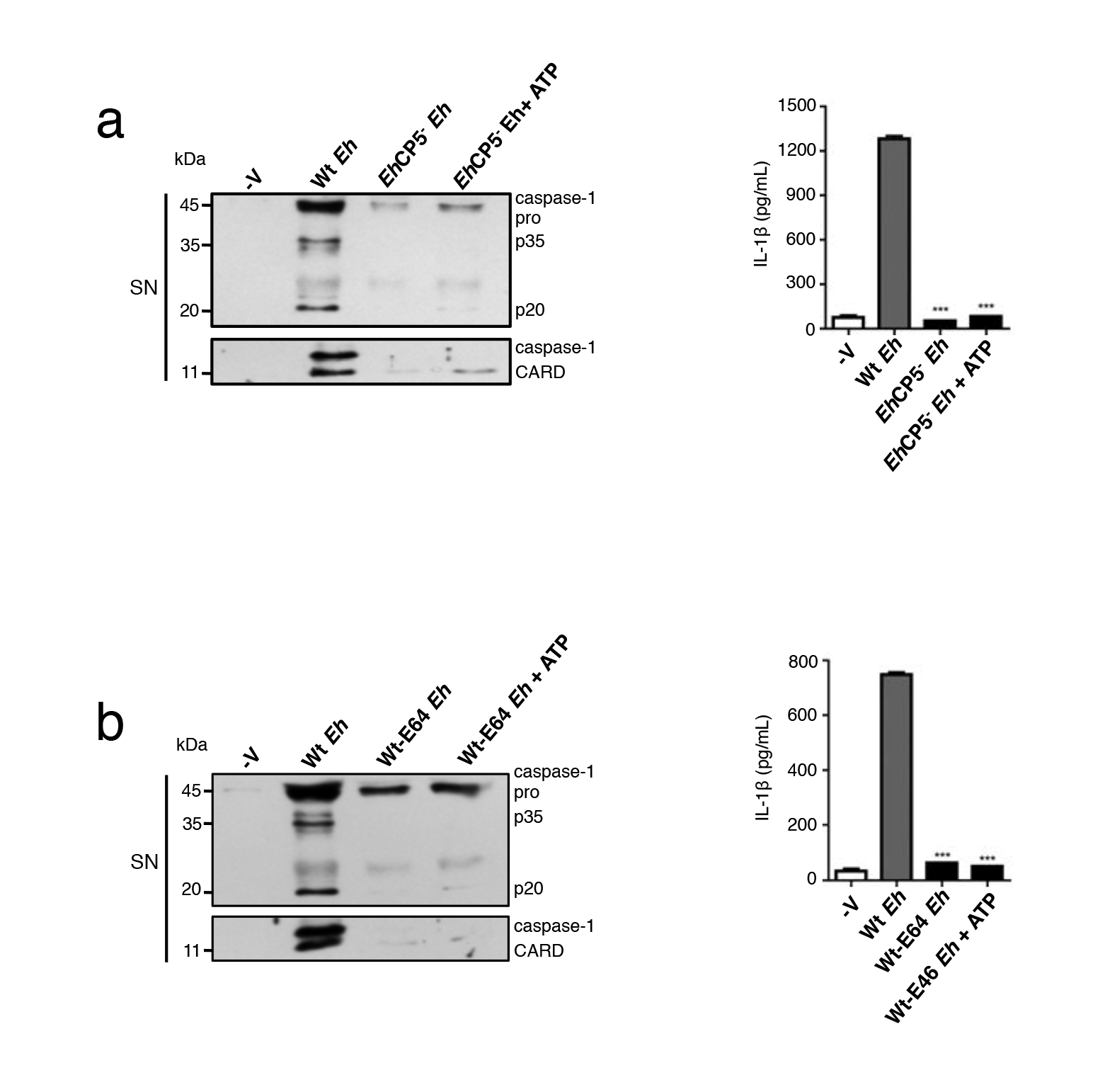

Supplement: S8 Fig — (a, b) Immunoblot analysis of secreted active caspase-1 cleavage products and IL-1β enzyme-linked immunosorbent assay of PMA-differentiated THP-1 macrophages stimulated for 30 min with Wt Eh or EhCP5- Eh (a) or Wt-E64 Eh (b) with or without the addition of ATP (5mM). Data are representative of three separate experiments (error bars SEM). (TIF) [file ppat.1004887.s008.tif]
